# The *Saccharomyces cerevisiae* Hot1p regulated gene *YHR087W* (HGI1) has a role in translation upon high glucose concentration stress

**DOI:** 10.1186/1471-2199-13-19

**Published:** 2012-06-21

**Authors:** M Gomar-Alba, E Jiménez-Martí, M del Olmo

**Affiliations:** 1 Departament de Bioquímica i Biologia Molecular, Facultat de Ciències Biològiques, Universitat de València, Dr. Moliner, 50. E-46100, Burjassot, Valencia, Spain; 2 Present address: Dept. Genètica, Fac. Biologia, Universitat de Barcelona, Barcelona, Spain

**Keywords:** *Saccharomyces cerevisiae*, High glucose osmotic stress, Gene *YHR087W*, Gene expression, Translation, Hot1p, Hog1p, Polysomes

## Abstract

**Background:**

While growing in natural environments yeasts can be affected by osmotic stress provoked by high glucose concentrations. The response to this adverse condition requires the HOG pathway and involves transcriptional and posttranscriptional mechanisms initiated by the phosphorylation of this protein, its translocation to the nucleus and activation of transcription factors. One of the genes induced to respond to this injury is *YHR087W*. It encodes for a protein structurally similar to the N-terminal region of human SBDS whose expression is also induced under other forms of stress and whose deletion determines growth defects at high glucose concentrations.

**Results:**

In this work we show that *YHR087W* expression is regulated by several transcription factors depending on the particular stress condition, and Hot1p is particularly relevant for the induction at high glucose concentrations. In this situation, Hot1p, together to Sko1p, binds to *YHR087W* promoter in a Hog1p-dependent manner. Several evidences obtained indicate Yhr087wp’s role in translation. Firstly, and according to TAP purification experiments, it interacts with proteins involved in translation initiation. Besides, its deletion mutant shows growth defects in the presence of translation inhibitors and displays a slightly slower translation recovery after applying high glucose stress than the wild type strain. Analyses of the association of mRNAs to polysome fractions reveals a lower translation in the mutant strain of the mRNAs corresponding to genes *GPD1*, *HSP78* and *HSP104*.

**Conclusions:**

The data demonstrates that expression of Yhr087wp under high glucose concentration is controlled by Hot1p and Sko1p transcription factors, which bind to its promoter. Yhr087wp has a role in translation, maybe in the control of the synthesis of several stress response proteins, which could explain the lower levels of some of these proteins found in previous proteomic analyses and the growth defects of the deletion strain.

## Background

Stress response in the yeast *Saccharomyces cerevisiae* involves the detection of adverse conditions (high or low osmolarity, nutrient limitation, ethanol exposure, increased level of oxidant reagents, variations in pH, etc.), activation of signal transduction pathways, and transcriptional and posttranscriptional regulation, resulting in the accumulation of protective agents and repairing activities [1]. All these mechanisms are intended to allow yeast cells to adapt to environmental changes.

Under several different stress conditions, *S. cerevisiae* displays a common response, the so-called *Environmental Stress Response* (ESR), characterized by changes in the expression of approximately 900 genes [2]. Most of these genes contain the AGGG consensus sequence in their promoter [3], which is recognized by transcription factors Msn2p and Msn4p [4]. The activity of these factors is regulated by two pathways that control cell growth: *Protein Kinase A* (PKA, [5]) and *Target of Rapamycin* (TOR, [6]).

Besides this general response, yeast cells display specific mechanisms to resist particular adverse conditions. In the case of hyperosmotic stress, produced by a high concentration of salt, sorbitol, glucose or of any other osmolyte, the response is mediated by the *High Osmolarity Glycerol* (HOG) pathway. High osmolarity is detected by osmosensors located in the membrane, that activate MAP quinases which, finally, permit the phosphorylation of MAPK Hog1p, which results in its translocation to the nucleus. Once inside this compartment, Hog1p activates several transcription factors (Hot1p, Msn1p, Smp1p, Gcn4p, Skn7p, Sko1p, Msn2p and Msn4p) [1,7,8]. These factors determine an induction in the expression of about 10% of the yeast genes under conditions of stress caused by high salt or sorbitol concentrations [2,9-12]. One of the consequences of these changes in gene expression is an increase in the intracellular concentration of glycerol, the osmolyte that yeast cells produce to counteract hyperosmotic stress.

One of the conditions of hyperosmotic stress that can affect yeast cells in particular environments (for instance, during the production of alcoholic beverages) is that produced by sugar concentrations of 20% (w/v) or even higher. Several transcriptomic analyses have been carried out to understand the particularities of the response to this form of stress [13-15]. All these studies have indicated that three groups of genes display higher expression levels under these conditions: i) genes involved in glycerol metabolism, ii) genes participating in response to chemical stimulus, and iii) several genes of unknown function. One of them, *YHR087W*, is induced approximately 5 times under 20% glucose and encodes for a protein of 111 amino acids (12 kDa). The expression of this gene also increases under other hyperosmotic stress conditions (salt and sorbitol) and in response to other adverse conditions such as heat shock, oxidative damage produced by H_2_O_2_ or diamide, ethanol, acid or basic pHs and the stationary phase [2,11,16]. The increase in the *YHR087W* mRNA levels under 20% glucose is also followed by higher content of the corresponding protein [15].

Some of the data obtained in recent years have demonstrated the relevance of the *YHR087W* gene expression in the response to high sugar concentrations. On the one hand, its overexpression in wine yeast strains results in an improved stress response and fermentative behavior [17]. Besides, strains with a high resistance to osmotic stress show higher mRNA levels corresponding to this gene [15]. Finally in laboratory strains, its disruption results in growth delay, lower viability and reduced glucose consumption under 25% and 30% glucose concentrations [15].

From the structural point of view, Yhr087wp presents a strong homology with the protein family related with human SBDS [18]. SBDS is the human protein whose mutation provokes the Shwachman-Bodian-Diamond syndrome, a rare autosomal recessive disorder with clinical features, including haematological dysfunction, pankreatic exocrine insufficiency and skeletal abnormalities, as well as a significant predisposition to the development of myelodysplasia and leukemia [19-21]. The yeast orthologue of SBDS is Sdo1p [22]. Yhr087wp contains the same structural elements as Sdo1p in the N-terminus region and, according to sequence analyses [18,23], they are distant homologues.

Sdo1p binds RNA, interacts with nuclear rRNA-processing factors [24], and is involved in the maturation of the ribosomal 60 S subunit required for the translational activation of ribosomes [25]. There is a report describing an aberrant regulation of Btn1p in the absence of Sdo1p, which suggests that portions of the ribosome maturation pathways survey the vacuolar function, presumably as a means to adjust protein levels for optimal cellular homeostasis [26]. Due to the structural relationship between Yhr087wp and Sdo1p, it has been proposed that the role of that protein in yeast cells would be associated in some way with the RNA metabolism [18]. Actually, synthetic lethality has been described between *yhr087w* and the mutants in genes encoding proteins involved in RNA processing and transport, translation and posttranslational processes ( *nat3* –acetylation of ribosomal proteins-, *nsr1* -synthesis of rRNA 18 S and its precursor 20 S-, *air1* –nuclear RNA processing-, *npl3* and *yra2p* -mRNA export [18]).

However, the data found by other authors suggest that Yhr087wp could be involved in other cellular processes. The *YHR087W* gene has also been named *RTC3* (from *Restriction of Telomere Capping*) because its null mutant suppresses the phenotype of termosensitive mutant *cdc13-1*[27]. In this mutant, at the non-permissive temperature, telomeric DNA is degraded and cell cycle progression is impaired. On the other hand, Costanzo et al. [28] described genetic interactions between *YHR087W* and several genes encoding proteins related with transcription and its control, such as Nut1p (a component of the RNA polymerase II mediator complex), Set3p (a member of a histone desacetylase complex), Npl3p (RNA polymerase II transcription elongation), Cna1p (a component of calcineurin) or Bcy1p (a regulatory subunit of the PKA). Finally, the results of a recent proteomic comparison made in our laboratory between deletion mutant *Δyhr087w* and its corresponding wild type strain [15] under 20% glucose showed lower levels of two proteins involved in protein folding (Hsp104p and Hsp78p) in the mutant strain, which appeared as an overrepresented category. In this sense, genetic interactions have been described between *yhr087w* and *hsp82*[29].

In this work we carry out several experiments in order to gain more insights into the role of Yhr087wp in yeast cells. The data obtained confirms once more the relationship between this protein and the stress response, provides new information about its transcriptional regulation, and points to a role in translation under adverse growth conditions.

## Results

### Yhr087wp is distributed throughout the cell

Data from a global study of protein localization by GFP fusions [30] indicate that protein Yhr087wp is distributed throughout the cell.

In order to verify this information and to determine whether the subcellular localization could be affected in some way when yeast cells are subjected to hyperosmotic stress conditions due to high glucose, a GFP tag was introduced into the chromosomal copy of gene *YHR087W* in the 3' coding region.

**Figure 1:**
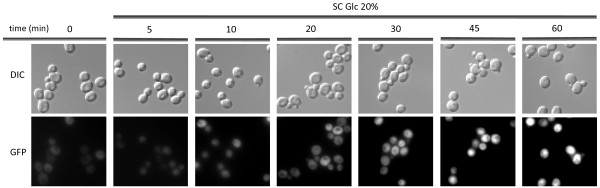
**Yhr087wp is distributed throughout the cell.** Fluorescence and DIC microscopy of cells expressing GFP-Yhr087wp under the control of the *YHR087W* promoter in its genomic localization. Cultures in the exponential growth phase in SC medium, without previous incubation in SC containing 20% (w/v) or at several time points after the transference to this medium were observed by fluorescence microscopy.

The fluorescence microscopy analysis of the exponentially growing cells (Figure 1) showed that Yhr087wp is located in the nucleus and cytoplasm, and that this location is not affected by changes in glucose concentration. Nonetheless, as previously described [15], an important increase in protein levels takes place when cells are transferred to SC 20% glucose. This increase is particularly important after 30 min.

### *YHR087W* is differentially regulated by transcription factors Msn2/4p and Hot1p depending on the stress conditions considered

**Figure 2:**
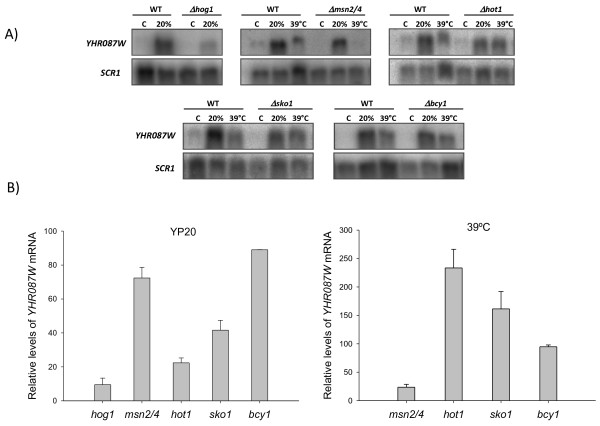
**Regulation of the*YHR087W*gene expression by the HOG pathway, the Msn2/4p, Hot1p and Sko1p transcription factors, and the Bcy1p subunit of the PKA.** Cells from the exponential cultures at 30°C in YPD medium of several wild type and mutant strains were maintained under these conditions (control, C), transferred to YP20 for 30 min (20%) or were affected by a 30-min heat shock at 39°C (39°C). Then were collected and total RNA was isolated. Gene expression was determined by Northern analysis using a specific probe. Data have been normalized with the *SRC1* gene. Images (panel **A**) show a representative example of the results obtained. Quantification of some of these results (panel **B**) has been carried out from the data obtained in three independent experiments; the average value and the standard deviation appear. In this quantification, the mRNA level found in each mutant under each stress condition is represented as the percentage of that detected in the corresponding wild type strain in the same situation, which is considered 100.

According to the microarray analyses carried out by Capaldi et al. [16], the increased expression of *YHR087W* under several conditions of osmotic stress is regulated through the HOG pathway. As this pathway is also involved in the response to high osmolarity due to 20% glucose [15] we determined if under this stress condition HOG1 controls *YHR087W* expression. For this purpose, cells exponentially growing in YPD medium from the strains described in Additional file 1: Table S1 TM141 (wild type) and TM233 (*hog1Δ*) were incubated for 30 min in YP20. RNA levels of gene *YHR087W* were determined by Northern analysis using a probe resulting from the amplification of genomic DNA by oligonucleotides YHR087W-1 and YHR087W-2 ( Additional file 1: Table S2). As shown in Figure 2*YHR087W* mRNA levels increase when cells are incubated for 30 min in growth medium containing 20% glucose (compare lanes C and 20% in WT in panel A), but the expression of the gene in the mutant is about ten times lower than in the wild type strain under these conditions (compare lanes 20% *Δhog1* and 20% WT and see the corresponding bar in panel B), thus demonstrating a role of the HOG pathway in the control of the transcription of this gene also when high glucose is used as an osmolyte.

The Msn2/4p transcription factors involved in the general stress response are activated by the HOG pathway under osmotic stress conditions. An analysis of the *YHR087W* sequence indicates the presence of STRE elements at positions -301 and -199, which could be recognized by these proteins. Besides, Msn2/4p proteins have been reported to be involved in the control of the *YHR087W* expression under certain conditions, such as zymolyase-induced cell wall stress [31], weak acid stress [32] and hyperosmolarity caused by high KCl concentrations [16]. To determine whether or not induction of *YHR087W* under high glucose concentrations follows the same pattern, RNA samples were obtained from exponentially growing cultures in YPD affected or not by 20% glucose or heat shock. These experiments were carried out with mutant strains in several of the transcription factors involved in the response to osmotic stress (Msn2/4p, Hot1p and Sko1p) using in each case its corresponding wild type strain as reference. As seen in Figure 2 panel A, the induction of this gene under heat shock occurs in all the wild type strains considered, and is only drastically reduced (to about 24% compared to its reference strain, panel B) in the *Δmsn2,4* mutant. However, under high glucose stress conditions, although the induction is also detected in all the wild type strains used in this analysis (panel A) the factors mainly involved in the regulation of the expression of this gene are Sko1p, and particularly Hot1p, being the reduction in the expression to about 40% and 20% respectively when compared to the reference strains (panel B).

**Figure 3:**
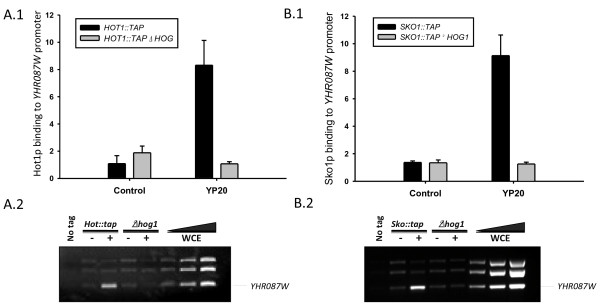
**Hot1p and Sko1p bind to the*YHR087W*promoter under high sugar stress in a Hog1p- dependent manner.** Panel **A1** shows the relative occupation of Hot1p over the region of the *YHR087W* promoter between -360 and -181, determined by chromatin immunoprecipitation (ChIP) followed by Real-Time PCR, in a Δ *hog1* mutant and its corresponding wild type strain, both of which contained a TAP-tagged version of Hot1p. Data were obtained as described in the *Materials and Methods* section. Experiments were carried out in triplicate; the average and standard deviation data are indicated. Panel **A2** contains the result of a representative ChIP experiment analyzed by semiquantitative PCR. In this case, a mix of three pairs of primers was used for each PCR reaction, which amplify the region corresponding to the *YHR087W* promoter described above and, those located between positions -750 and -420 of gene *CLN2* and -822 to -567 of gene *CLB2*. In this panel WCE indicates the whole cell extract prior to the inmunoprecipitation. For these experiments, cells from the exponentially growing cultures were also incubated for 10 min in YP20 (YP20 in panel A.1, + in panel A.2) or not (Control in panel A.1, - in panel A.2) to determine the dependence of Hot1p binding on high glucose stress. Panels **B1** and **B2** correspond to the same experiments in the case of a Δ *hog1* mutant and its corresponding wild type strain, both of which contained a TAP-tagged version of Sko1p.

Chromatin immunoprecipitation experiments carried out with a wild type strain and its corresponding *Δhog1* mutant both containing a Hot1-TAP tagged version (Figure 3 panel A) indicate that Hot1p binds to a region of the *YHR087W* gene promoter located between positions -360 and -181 under high glucose stress conditions and that this interaction depends on the presence of Hog1p. Accordingly, the involvement of Hot1p in the regulation of *YHR087W* expression under 20% glucose is similar to that found for other osmostress-induced genes, such us *CTT1HSP12* and *STL1*, with exposure to NaCl 0.4 M [33]. Similar experiments have been carried out with the corresponding strains containing a Sko1p-TAP tagged version (Figure 3 panel B) and demonstrate that this transcription factor also binds to the region considered of *YHR087W* promoter in a Hog1p dependent manner.

### Role of Yhr087wp in transcription and its control

Costanzo et al. [28] created a genome-scale genetic interaction map by examining 5.4 million gene-gene pairs for synthetic genetic interactions. With this approach, these authors described the interactions between *YHR087W* and several genes related to transcription and its control, such as *BCY1CNA1NUT1SET3*, and *NPL3*. In order to corroborate these interactions and to determine their relevance, growth of serial dilutions of yeast cells in YPD solid medium at 37°C, or containing 30% glucose, was determined. This glucose concentration was selected according to previous data obtained in our laboratory [15] and to the results shown in Additional file 2: Figure S1, which indicate that a growth defect occurs in strain *Δyhr087w* at this glucose concentration, which can be followed by growth in such plates. For these experiments, we focused on the determination of the relationship between *YHR087W* and *BCY1* (coding for the regulatory subunit of the PKA) and *CNA1* (which encodes for calcineurine). For this purpose *YHR087W* disruption was carried out in mutant strains in all of those genes.

**Figure 4:**
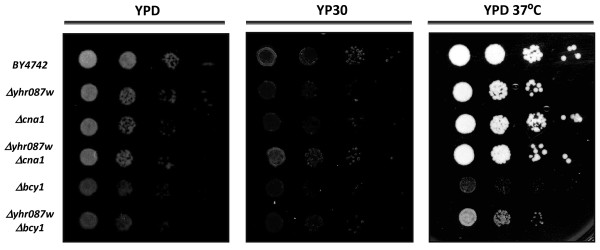
**Genetic interactions between*YHR087W*and*BCY1*and*CNA1*.** Growth of mutants Δ *yhr087w*, Δ *bcy, yhr087wbcy, cna1* and *Δyhr087wΔcna1* under osmotic (30% glucose) and heat shock (37°C) stress. Serial dilutions from the cultures of these strains were spotted and growth was followed for 3 days at 30°C or 37°C, as described in the *Materials and Methods* section.

Regarding gene *CNA1*, it was not possible to detect any genetic relationship under 37°C or at a high glucose concentration (Figure 4), maybe because of the different growth conditions (compared to those used by Constanzo et al. [28]) used in this work in order to get high expression of gene *YHR087W*. However, with gene *BCY1*, a partial supression of its growth defects at 37°C was detected in the double mutant with *Δyhr087w*. As shown in Figure 4, a double mutant grows better than the *Δbcy1* single mutant at 37°C. Despite this interaction, the *YHR087W* expression is not affected in the *Δbcy1* mutant strain under heat shock stress or at high glucose concentrations (Figure 2). Being Bcy1p the regulatory subunit of PKA, this result reinforces the Yhr087wp’s involvement in the stress response.

The genetic interactions commented above suggest that this protein could indeed play some direct role in transcription. ChIP analyses were carried out to test the possible binding of Yhr087wp to DNA, particularly to the stress-induced gene *HSP104*. For this purpose, a short incubation at 37°C, or under 20% (w/v) glucose, was applied to the exponentially growing cells carrying fusion protein Yhr087w-TAP, created by genetic manipulation in the chromosomal copy of gene *YHR087W*, as described in the *Materials and Methods* section. The results indicate that Yhr087wp does not bind to any of the regions considered in *HSP104* DNA under any of the study conditions because the absolute values are very similar to those found under the same conditions with a non labeled strain used as a negative control ( Additional file 2: Figure S2). Additional ChIP experiments described in the Materials and Methods section indicated that the binding of the RNA polymerase II to *HSP104* promoter is not affected in the *Δyhr087w* mutant (data not shown).

### Yhr087wp interacts with proteins involved in stress response and ribosomal function

Of the various experimental methods available for identifying protein-protein interactions, this work considers the tandem affinity purification (TAP) of TAP-tagged Yhr087p expressed from its natural chromosome location (strain BY4741 *YHR087W:: TAP:: kanMX4*) to carry out a study on this protein.

In the global analysis of the protein complexes developed a few years ago by Krogan et al. [34], Yhr087wp appeared to physically interact with Eki1p (ethanolamine kinase) and Suc2p (invertase). An interaction with ribosomal protein Rpp0p was previously described [35]. As these data were obtained under non stress growth conditions, a TAP purification experiment was carried out after a 90-min incubation in YP20 of YPD-growing cells. Previous experiments carried out in our group [15] indicated that protein levels significantly increased under this condition when compared to control growth.

The analysis showed 45 proteins with the MASCOT software (Mascot Daemon, Matrix Science) that fulfill the conditions so they could be considered candidates for the interaction with Yhr087wp, whereas this number rose to 138 when the Protein Pilot software (AB Sciex), version 2.0 was used. After excluding from the analysis the proteins that appear with a high frequency in successful protein purifications with this procedure ([34]; Pamblanco, personal communication, Rodriguez-Navarro, personal communication) a total of 10 proteins, that are coincident in both tests, were finally considered (Table 1) to be potential Yhr087wp-interacting proteins. Most of the proteins identified belong to two categories: stress response and translation. Actually, according to the FuncAssociate tool (http://llama.mshri.on.ca/funcassociate/) two statistically significant categories emerge: calmodulin-dependent protein kinase activity (with a p-value of 0.000003438) and eukaryotic translation 4F complex initiation factor (with a p-value of 0.00003427). These results demonstrate once again the relationship between Yhr087wp and stress response. Besides, the detection of interactions with factors involved in translation initiation (such as eIF4E, eIF4G and eIF3) suggests a putative role for the ribosomal function.

**Table 1:** Yhr087wp interacting proteins according to the TAP experiments carried out in this work

**Protein**	**Function**^**(1)**^
Atp7p	Subunit d of the stator stalk of mitochondrial F1F0 ATP synthase
Osh7p	Member of an oxysterol-binding protein family with seven members in *S. cerevisiae*; family members have overlapping, redundant functions in sterol metabolism and collectively perform a function essential for viability
Cmk1p	Calmodulin-dependent protein kinase; may play a role in stress response
Cmk2p	Calmodulin-dependent protein kinase; may play a role in stress response
Sti1p	Hsp90 cochaperone, interacts with the Ssa group of the cytosolic Hsp70 chaperones and activates Ssa1p ATPase activity
Tfg1p	TFIIF (Transcription Factor II) largest subunit; involved in both transcription initiation and elongation of RNA polymerase II;
Cdc33p	Cytoplasmic mRNA cap binding protein and translation initiation factor eIF4E; the eIF4E-cap complex is responsible for mediating cap-dependent mRNA translation via interactions with translation initiation factor eIF4G (Tif4631p or Tif4632p)
Scp160p	Essential RNA-binding G protein effector of mating response pathway, mainly associated with nuclear envelope and ER, interacts in mRNA-dependent manner with translating ribosomes via multiple KH domains, similar to vertebrate vigilins
Tif32p	eIF3a subunit of the core complex of translation initiation factor 3 (eIF3), essential for translation
Tif4631p	Translation initiation factor eIF4G, subunit of the mRNA cap-binding protein complex (eIF4F) that also contains eIF4E (Cdc33p); interacts with Pab1p and with eIF4A (Tif1p); also has a role in biogenesis of the large ribosomal subunit

^(1)^ The description according to *Saccharomyces Genome Database* (SGD) is shown.

**Figure 5:**
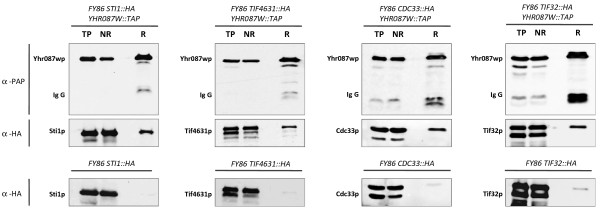
**Demonstration of the physical interaction between Yhr087wp, Sti1p, Tif4631p, Cdc33p and Tif32p by coimmunoprecipitation.** Cells of the strains carrying a TAP-tagged version of the Yhr087wp and an HA-tagged version of Sti1p, Tif4631p, Cdc33p or Tif32p proteins were exponentially grown in YPD at 30°C. After a 90-minute incubation in YP20 at the same temperature, cells were lysed and Yhr087p was immunoprecipitated as described in the *Materials and Methods* section. This Figure shows the Western analysis carried out with the total protein samples (TP), the non-retained fraction (NR) and the retained fraction (R). Yhr087wp is detected with α-PAP antibody, while the other four proteins were visualized with α-HA antibody. The results of the control experiments carried out with a FY86-derived strain carrying HA-tagged versions of the Sti1p, Tif4631p, Cdc33p and Tif32p proteins are also shown.

The co-immunoprecipitation experiments carried out with those strains carrying the TAP-tagged version of Yhr087wp and an HA-tagged version of Sti1p, Tif4631p, Cdc33p or Tif32p confirm the interactions found with the standard TAP protocol (Figure 5). As this Figure depicts, these proteins appear in the retained fraction after precipitation of the TAP-tagged protein with the α-PAP antibody.

**Figure 6:**
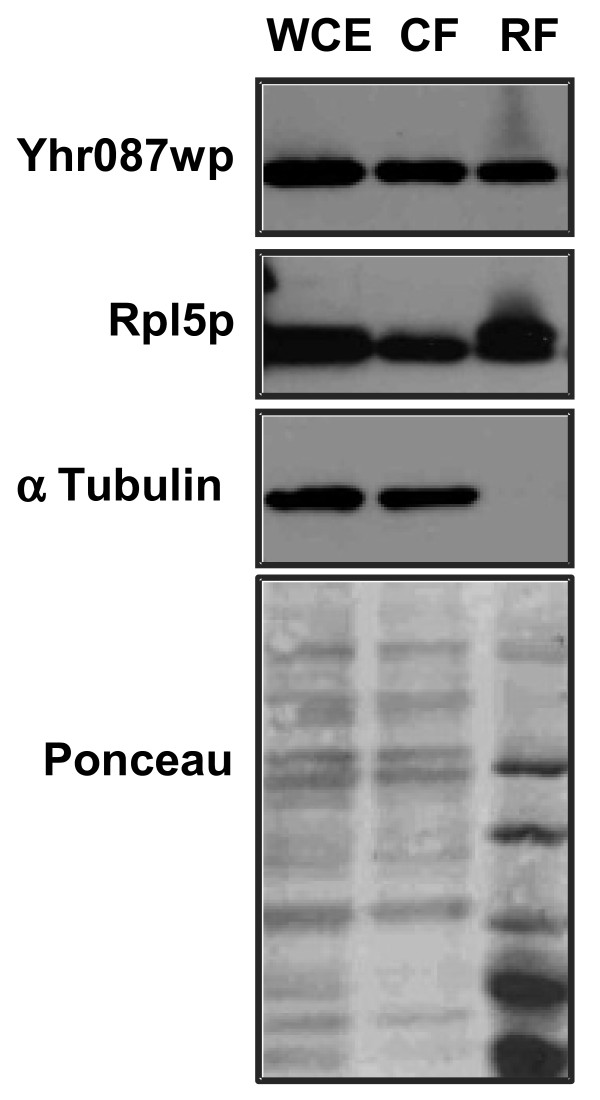
**Yhr087wp partially associates with ribosomes.** Whole cell extracts obtained from a strain containing an Yhr087wp-TAP-tagged protein were fractionated as described in the *Materials and Methods* section. The Figure shows the distribution of protein Yhr087wp between the cytoplasmic (CF) and ribosomal (RF) fractions by Western analysis. A sample corresponding to the whole cell extract (WCE) is also included. As a control of the fractionation procedure, the location of a cytosolic (α-tubulin) and a ribosomal protein (Rpl5p) was determined by Western analysis using specific antibodies against these proteins.

Ribosome isolation experiments and detection of Yhr087wp protein location by Western blot have indicated that this protein is found, at least partially, in the ribosomal fraction (Figure 6). In these experiments, two control proteins were considered: α subunit of tubulin, which is completely found in the non ribosomal fraction, and the ribosomal protein Rpl5p, mainly detected in the ribosomes.

### The *YHR087W* deletion mutant shows an increased sensitivity to translation inhibitors

The TAP experiment results suggest the possible implication of Yhr087wp in the ribosomal function. To gain more evidences about this hypothesis, sensitivity to translation inhibitors was determined in the deletion mutant of the coding gene. Two drugs were considered for this purpose: cycloheximide (which interferes with the peptidyl-transferase activity) and hygromycin (which stabilizes the tRNA ribosomal acceptor site, thus preventing translocation). These inhibitors were tested not only with this strain, but also with mutants in *STI1* and in genes encoding the proteins involved in translation which appeared as candidates for the interaction with Yhr087wp according to the TAP analysis. Besides, double mutant strains with each one of these genes and *YHR087W* were obtained as described in the Materials and methods section and were considered for this analysis.

**Figure 7:**
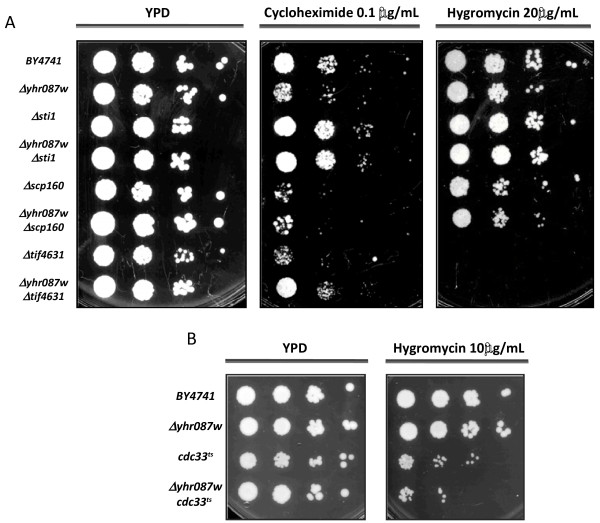
**Sensitivity to translation elongation inhibitors of the mutants in*YHR087W*and/or other genes encoding proteins that physically interact with Yhr087wp (according to the TAP experiment) and are involved, most of them, in the ribosomal function:*STI1*,*SCP160*,*TIF4631*(Panel A) and*CDC33*(Panel B).** Serial dilutions were spotted on YPD, YPD cycloheximide (0.1 μg/mL) and YPD hygromycin (20 μg/mL or 10 μg/mL) plates, which were incubated for 48-72 h at 30°C. Incubation with mutants *cdc33*^*ts*^ and *Δyhr087w cdc33*^*ts*^ was carried out at 33°C until growth was detected.

As Figure 7A shows, the mutants in the genes somehow related with the ribosomal function display sensitivity to one and/or another drug. With *Δscp160*, high sensitivity to cycloheximide was found, while growth was affected to a lesser extent by the presence of hygromycin. *Δtif4631* displayed total lack of growth in hygromycin, and very pronounced growth defects in cycloheximide. Growth in the presence of these drugs was not affected, however, by the deletion of the gene *STI1*, which does not participate in translation. With the *Δyhr087w* mutant, sensitivity to cycloheximide was greater than to hygromycin. The opossite result was described for mutants in *SDO1* such as *sdo1*-K118A [25]. The introduction of the *YHR087W* deletion into these backgrounds did not result in enhanced or disminished sensitivity to the inhibitors tested.

Experiments were also carried out with thermosensitive mutants in *TIF32* and in *CDC33* and the corresponding double mutants with *Δyhr087w*. In the case of *tif32*^*ts*^ no sensitivity was detected to the inhibitors under the conditions considered and no additional effect was displayed by the double mutant (data not shown). For *cdc33*^*ts*^, as can be seen in Figure 7, the sensitivity to hygromycin of the single mutant in translation initiation factor eIF4E when incubated at the non permissive temperature at a concentration of 10 μg/mL increased in the double mutant with *Δyhr087w*. For a more accurate determination of the differences between these single and double mutants, the number of colony forming units and the viability under this concentration of hygromycin were determined (Table 2). The data obtained confirms the results of the serial dilution analysis and indicates that the variations between strains are statistically significant. It is worth mentioning that similar experiments were also carried out in plates contatining 20 μg/mL of hygromycin; in this case the viability was very low being four times lower in the double mutant than in the single *cdc33*^*ts*^ (1% compared to 4%).

**Table 2:** **Viability of single and double mutant strains in*YHR087W*and*CDC33*under hygromycin 10 μg/mL**

**Strain**	**Viability**
BY4741	95,33 ± 8,08
*Δyhr087w*	73,67 ± 0,58
*cdc33*^*ts*^	41,71 ± 8,33
*Δyhr087w cdc33*^*ts*^	27,34 ± 3,45

Dilutions from cultures in exponential phase of growth were plated on YPD or YPD + hygromycin 10 μg/mL plates and the colony forming units appeared 48-72 hours later were counted. Viability was determined as the percentage between the CFU in hygromycin plates and in YPD plates. Application of the *t*-test to the data obtained for *cdc33*^*ts*^ and *Δyhr087w cdc33*^*ts*^ mutant strains gave a *P* value (calculated for one tail using Microsoft Office Excel) of 0.0025.

The sensitivity to cycloheximide of the *Δyhr087w* mutant and the interaction between Cdc33p and Yhr087wp provide new insights about the role of this protein in translation.

### Translation recovery after osmotic stress due to high glucose concentrations is slightly affected in the *Δyhr087w* mutant

The separation of the fractions of the translation complexes associated with mRNAs by sucrose gradients is a powerful methodology to study of global protein synthesis and the physiological functions of the individual factors involved in this process [36]. The analysis of the polysome profiles has also shown that when yeast cells are affected by high salinity stress, a temporary shutdown of translation in short times occurs, followed by a subsequent recovery in time [37].

To determine whether increased sensitivity to the translation inhibitors detected in strain *Δyhr087w* is associated with defects in this process, we analyzed the polysome profiles of this mutant and the corresponding wild type strain. For these experiments, the exponentially growing cultures of these strains were transferred to an OD_600_ of 0.4 to YP20. Samples were collected at different times to analyze the changes in the translational activity noted during the first minutes of incubation under 20% glucose, as well as the effect of the mutation in gene *YHR087W*. The time 0 sample was obtained before the transfer to the high glucose concentration medium took place.

**Figure 8:**
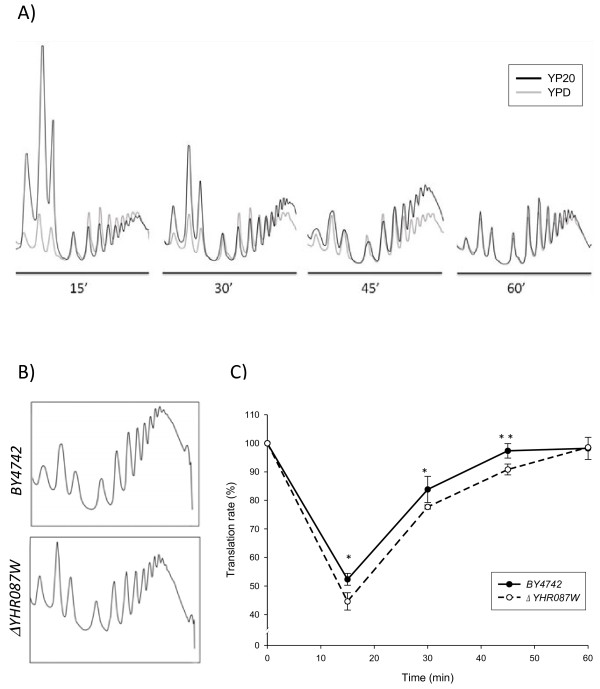
**Polysome profiles in the wild type (BY4742) and*Δyhr087w*strains suggest an involvement of protein Yhr087wp in the translation process.** The overlapping polysome profiles found for the wild type strain at several timepoints during the first hour after addition of 20% (w/v) glucose to the exponentially growing cells compared to the previous situation (panel **A**). Comparison made between the polysome profiles of the wild type and mutant strains at 45 min after introducing high sugar stress (panel **B**). Percentage of the transcription rate (relative to time 0) found for both strains during the time course of the experiment (panel **C**). In this panel asterisks indicate statistically significant differences (* p-value < 0.05, ** p-value < 0.005). More details about the experiment are included in the *Materials and Methods* section.

Figures 8A and B show the polysome profiles obtained in these experiments. In order to gain a better understanding of the information provided by our analyses, Table 3 presents the percentage of area corresponding to the polysomal fraction in the profiles. From these data, it is possible to determine the relationship between the polysome area (P) under stress (P_S_) and under normal growth conditions (P_N_). This parameter represents the relative change in mRNA associated with the ribosomes participating presumably in translation [37]. The values in Table 3 also allow us to determine the relationship between the nonpolysomal (free and monosomal, FM) fraction under both conditions (FM_S_/FM_N_), which represents the relative change in mRNA that is not translated. If we consider all these data, it is possible to estimate the percentage of translation, determined as the relationship of the polysomal fraction between a particular timepoint compared to the time of the stress application (Figure 8C).

**Table 3:** **Analysis of the polysome profile obtained for the wild type and the*Δ*yhr087wstrains**

	**time (min)**	**0**	**15**	**30**	**45**	**60**
BY4742	P (1)	77.12 ± 3.67	40.37 ± 1.60	64.65 ± 3.54	75.07 ± 1.95	75.74 ± 2.98
	P/(F + M)(2)	3.43 ± 0.71	0.68 ± 0.04	1.84 ± 0.28	3.03 ± 0.33	3.15 ± 0.51
	60/40 (3)	1.49 ± 0.19	1.73 ± 0.13	1.37 ± 0.39	1.23 ± 0.06	1.55 ± 0.26
Δ*yhr087w*	P (1)	76.34 ± 2.89	34.06 ± 2.31	59.32 ± 0.55	69.33 ± 1.46	75.25 ± 0.01
	P/(F + M) (2)	3.26 ± 0.52	0.52 ± 0.05	1.46 ± 0.03	2.27 ± 0.16	4.54 ± 0.00
	60/40 (3)	1.23 ± 0.14	1.66 ± 0.05	1.52 ± 0.28	1.15 ± 0.12	1.63 ± 0.12

(1) The data shown correspond to the percentage of the polysome area relative to the total area of polysomal plus free plus monosomal fractions. The amount of rRNA in each fraction was measured by UV detection at 260 nm. Three independent experiments were carried out: average and standard deviation are shown.

(2) Ratio between the area of the polysomal and free plus monosomal fractions.

(3) Ratio between the area corresponding to the 60 S and 40 S subunits.

As shown in Figure 8A, the introduction of osmotic stress due to high glucose concentration determines a similar effect in the wild type yeast cells to that described for 1 M NaCl by Melamed et al. [37]. Translation markedly lowers during the first minutes, which can be clearly detected at 15 min. At this time, the translation rate drops almost a half (Figure 8C), and the P_S_/P_N_ ratio is 0.52, while the FM_S_/FM_N_ ratio shows a value of 2.6. Melamed et al. [37] revealed descreases in the polysomal fraction, which are higher for 1 M NaCl (around 3.5 times). Thirty min after applying the high glucose concentration, the wild type strain is capable to recover around 84% of the initial level of translation, and the situation is very similar to that before applying stress (97%) after 45 min, suggesting that the translational effect of this kind of osmotic stress is lower than that provoked by high salinity (more than 3 h, [37]).

For strain *Δyhr087w*, a slightly higher decrease in translation was noted at 15 min after introducing the high sugar stress, with values for the P_S_/P_N_ and FM_S_/FM_N_ ratios of 0.45 and 2.79, respectively. Besides, as shown in Figure 8B for the 30 and 45 min timepoints, the recovery of translation shows a delay when compared to the wild type strain (78% at 30 min and 91% at 45 min). After 60 minutes, this strain had already recovered the initial levels of translation.

Although the differences between mutant and wild type strains are low, they are statistically significant at time points 15, 30 and, particularly, 45 min, which provides another result that points to an involvement of Yhr087wp in the ribosomal function. Unlike the results described for the *SDO1* mutants [25], this role would not seem to be related to the maturation of the 60 S subunit, as the ratio of the areas corresponding to 60 S/40 S fractions (Table 3) does not consistently decrease in mutant *Δyhr087w*.

### The translation of several mRNAs is affected in the *Δyhr087w* deletion mutant under high glucose stress conditions

According to the results of a proteomic analysis [15], levels of several proteins (Hsp104p among them) in the *Δyhr087w* deletion mutant are lowered at the 20% glucose concentration. Additional file 2: Figure S3 confirms these results and indicates that the amount of this protein did not increase at 1 h after applying high glucose stress to exponential cultures of this mutant. These differences in protein levels cannot be explained by transcriptional control because the *HSP104* mRNA levels (relative to *ACT1*) under this stress condition, determined by Real-Time RT-PCR, are 5.55 and 5.51, respectively.

**Figure 9:**
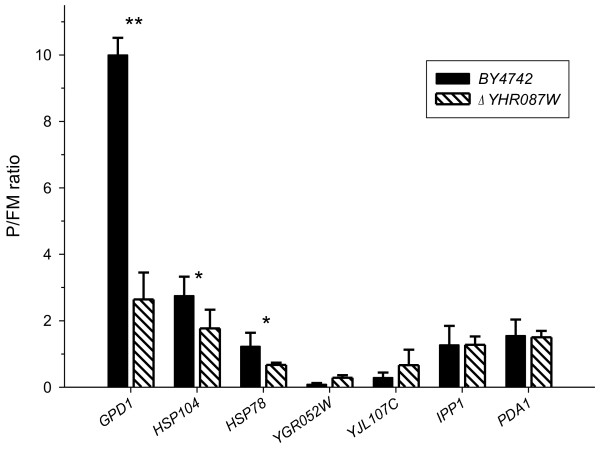
**The translation of several stress-induced genes is affected in strain*Δyhr087W*.** Cultures of the WT and Δ *yhr087W* strains growing exponentially in YPD were incubated for 45 min in 20% glucose and polysomal profiles were obtained. The fractions corresponding to free and monosomal (FM) and polysomal (P) were collected, RNA was isolated and cDNA was obtained. The expression of the indicated genes was analyzed by Real-Time RT-PCR using specific probes. Signals were normalized to *Bacillus subtilis LysA* mRNA, which was added to each group of fractions in equal amounts. The expression values shown are relative to those found in the *ACT1* gene. The quantified signals from three independent replicates were used to determine the average change in P/FM ratios. Asterisks indicate statistically significant differences (* p-value < 0.05, ** p-value < 0.005).

The slightly lower translation recovery found in the *Δyhr087w* mutant strain after applying the high glucose stress prompted us to determine if the distribution of *HSP104* mRNA between the polysomal and monosomal fractions could be affected in this strain. As shown in Figure 9, the P/FM ratio, is 1.5 times lower in the *Δyhr087w* mutant than in the corresponding wild type strain at 45 min after applying the adverse condition. This difference is in the threshold to be considered statistically significant (p-value of 0.049), is consistently found in all the replicates of the experiment and could explain the differences noted in the Hsp104p levels between these two strains in the proteomic and Western blot analyses. This difference in mRNA translation is more clear and significant from the statistical point of view in the case of genes coding for the proteins involved in the stress response *HSP78* (p-value of 0,033) and, particularly, *GPD1* (p-value of 0,002).

Figure 9 indicates that not all the mRNAs in this mutant are affected in the same way. Genes *IPP1* and *PDA1* do not display differences in their P/FM ratio. Moreover, two other genes induced by osmotic stress because of high glucose concentrations, *YGR052W* and *YJL107C*[13-15], appear to be better translated in the mutant strain at this timepoint, although without statistically significant differences.

### Yhr087wp has homologues in other yeasts

The application of the BLASTP tool has allowed the identification of Yhr087wp homologous proteins in many yeasts and fungi (Table 4). So far little is known about these proteins and their role, but global transcription analyses carried out in *Schizosaccharomyces pombe*[38] have shown that the ortholog in this microorganism (SPBC21C3.19) is induced also by several stress conditions, including osmotic stress (1 M sorbitol), heat shock (39 deg;C), and oxidative injuries (0.5 mM H_2_O_2_).

**Table 4:** **Yhr087wp homologue proteins**^**1**^

**Accession number**	**Protein**	**Max score**	**Total score**	**Query coverage**	**E-value**
NP_011955	Rtc3p (*Saccharomyces cerevisiae* S288c)	221	221	100%	5e-74
XP_002494671	ZYRO0A06974p (*Zygosaccharomyces rouxii*)	133	133	89%	3e-39
XP_001646516	Hypothetical protein Kpol_1055p14 (*Vanderwaltozyma polyspora* DS 70294)	121	121	88%	1e-34
XP_446886	Hypothetical protein (*Candida glabrata* CBS 138)	120	120	89%	4e-34
XP_456050	Hypothetical protein (*Kluyveromyces lactis* NRRL Y-1140)	119	119	99%	7e-34
CCC71492.1	Hypothetical protein NCAS_0H01820 (*Naumovozyma castellii* CBS 4309)	108	108	81%	1e-29
XP_002553632	KLTH0E03454p (*Lachancea thermotolerans*)	106	106	80%	1e-28
CCD23842.1	Hypothetical protein NDAI_0C01810 (*Naumovozyma dairenensis* CBS 421)	101	101	76%	2e-26
XP_002615723	Hypothetical protein CLUG_04605 (*Clavispora lusitaniae* ATCC 42720)	68.2	68.2	89%	1e-13
EGV64915.1	Hypothetical protein CANTEDRAFT_121094 (*Candida tenuis* ATCC 10573)	67	67	86%	4e-13
XP_001212546	Conserved hypothetical protein (*Aspergillus terreus* NIH2624)	60.1	60.1	83%	3e-10
NP596599	DUF1960 family protein (*Schizosaccharomyces pombe* 972 h-)	59.3	59.3	77%	4e-10
XP_001386005.2	Hypothetical protein PICST_36865 (*Scheffersomyces stipitis* CBS 6054)	57.8	57.8	80%	1e-09
XP_001804429	Hypothetical protein SNOG_14233 (*Phaeosphaeria nodorum* SN15)	58.2	58.2	90%	1e-09
EFW98381.1	Hypothetical protein HPODL_0061 (*Pichia angusta* DL-1)	58.2	58.2	80%	2e-09
XP_461344.1	DEHA2F23078p (*Debaryomyces hansenii* CBS767)	57	57	81%	3e-09
XP_713165.1	Hypothetical protein CaO19.9418 (*Candida albicans* SC5314)	57	57	89%	4e-09
XP_001935065.1	Conserved hypothetical protein (*Pyrenophora tritici-repentis* Pt-1 C-BFP)	57	57	83%	4e-09
EDK_36318.2	Hypothetical protein PGUG_00416 (*Meyerozyma guilliermondii* ATCC 6260)	56.6	56.6	94%	6e-09
XP_001525927.1	Conserved hypothetical protein (*Lodderomyces elongisporus* NRRL YB-4239)	56.6	56.6	75%	6e-09
XP_001819636.1	RNA binding protein (*Aspergillus oryzae* RIB40)	56.2	56.2	89%	7e-09
XP_002418668.1	Protein involved in RNA metabolism, putative (*Candida dubliniensis* CD36)	55.8	55.8	88%	1e-08
XP_001594882.1	Hypothetical protein SS1G_04690 (*Sclerotinia sclerotiorum* 1980)	55.5	55.5	76%	1e-08
XP_002543973.1	Conserved hypothetical protein (*Uncinocarpus reesii* 1704)	54.7	54.7	85%	2e-08
XP_001393111.1	RNA binding protein (*Aspergillus niger* CBS 513.88)	54.7	54.7	82%	3e-08
XP_003347541.1	Hypothetical protein SMAC_04847 (*Sordaria macrospora* k-hell)	54.3	54.3	84%	4e-08
EGW33008.1	Hypothetical protein SPAPADRAFT_60332 (*Spathaspora passalidarum* NRRL Y-27907)	54.3	54.3	80%	4e-08
XP_003296114.1	Hypothetical protein PTT_04921 (*Pyrenophora teres* f.teres 0-1)	54.3	54.3	81%	8e-08
EGO53727.1	Hypothetical protein NEUTE1DRAFT_119291 (*Neurospora tetrasperma* FGSC 2508)	53.1	53.1	75%	1e-07
XP_964260.1	Hypothetical protein NCU02765 (*Neurospora crassa* OR74A)	52.4	52.4	75%	2e-07
CBX97830.1	Similar to RNA binding protein (*Leptosphaeria maculans*)	52	52	83%	3e-07
XP_002793737.1	Conserved hypothetical protein(*Paracoccidioides brasiliensis* Pb01)	50.8	50.8	82%	8e-07
XP_001560959.1	Hypothetical protein BC1G_00044 (*Botryotinia fuckeliana* B05.10)	50.8	50.8	86%	8e-07
XP_002564167.1	Pc22g01230 (*Penicillium chrysogenum* Wisconsin 54-1255)	50.8	50.8	74%	9e-07

^1^BLASTP2.2.25 was used for the search [39,40]. Data show only the homologue proteins with E-values lower than exp-07 from a total of 84 blast hits. All the non-redundant GenBank CDS translations + PDB + SwissProt + PIR + PRF, excluding the environmental samples from WGS projects, were considered.

The sequence alignment among some of these homologue proteins ( Additional file 2: Figure S4) shows four particularly conserved regions located between positions 30-53, 63-75, 80-98 and 102-114. Residues K34, V42, V67, V72, F73, A88, V93, F97, G98, E105, V106, T110, L111 and G114 are identical or conserved with SBDS proteins [18].

## Discussion

In this work we carry out a functional analysis of gene *YHR087W*. To date this gene has been related to many different cellular processes, and determining its main role in yeast cells is proving to be a difficult task. The results previously obtained in our laboratory indicate an involvement of Yhr087wp in the osmotic stress caused by high glucose concentrations, although the gene expression data published by other authors suggest that it may also participate in the response to other forms of stress [2,11]. The link between Yhr087wp and response to stress (particularly osmotic stress caused by high glucose concentrations) is based on the following aspects: i) deletion of the coding gene results in a lower growth rate in the presence of 25% and 30% glucose concentrations [15] ii) wine yeast strains displaying better tolerance to osmotic stress have high levels of the mRNA of this gene [15]; iii) its overexpression improves the osmotic stress resistance and fermentative behavior of these strains [17]; iv) the proteomic study carried out with the *Δyhr087w* deletion mutant [15] provides a strong link between the levels of this gene expression under high glucose concentrations and the Hsp104p and Hsp78p levels; v) genetic interactions between *YHR087W* and *HSP82*, another gene encoding an Hsp protein [29] have been described. This work demonstrates another interesting interaction which was previously suggested by Constanzo et al. [28]: *Δyhr087w* is capable of partially suppressing the growth defects displayed by the *Δbcy1* mutant at 37°C. As Bcy1p encodes the regulatory subunit of Protein Kinase A, involved in the control of cell growth and stress response, the connection of Yhr087p with this process becomes more evident.

On the other hand, results shown in Figure 2 demonstrate that the expression of *YHR087W* is controlled mainly by Msn2/4p transcription factors under heat shock, and by Hot1p and Sko1p (in a HOG-dependent manner) when osmotic stress due to high glucose concentration is applied. This differential regulation depending on the stress conditions could be explained by inhibition under 20% glucose of Msn2/4 activity and hence by an overall decrease in the activation of the general stress response in these conditions, according to Capaldi et al. [16].

Once the relationship between Yhr087wp and stress response is established, the question posed would be how this protein is involved in it and why is there a lower expression of several stress response proteins in this mutant. One possibility is that it would develop some transcriptional (by binding to the chromatin or by participating in the RNA polymerase II recruitment) or posttranscriptional (mRNA export or stability, for instance) role. This could explain the described genetic interactions with Mdm20p/Nat3p, Nsr1p, Npl3p, Yra2p and Air1p [18]. However, the transcriptomic experiments carried out in our laboratory (Jiménez-Martí and del Olmo, unpublished results) indicate a very low number of genes that are transcriptionally affected in the *Δyhr087w* mutant (38 genes more expressed in the mutant and 8 in the wild type strain) and they cannot be included in any statistically significant category. Besides, other studies also carried out by our group indicate that Yhr087wp is not involved in the mRNA export (Jiménez-Martí and del Olmo, unpublished results), the binding to yeast promoters ( Additional file 2: Figure S2), the recruitment of RNA polymerase II to them or the stability of the messengers (data not shown).

The results obtained in the TAP experiments described in this work seem to assign a protein translation-related function to Yhr087wp. The physical interactions found in these experiments with several of the factors involved in translation (Table 1), its partial association with the ribosomal fraction (Figure 6), the fact that the deletion mutant displays lower levels of proteins associated with ribosomal function [15], and the genetic interactions noted in this work (Figure 7) with the gene encoding translation initiation factor eIF4E (Cdc33p) and those described by other authors with Nat3p and Nsr1p [18], all support this potential role of the protein. It is worth mentioning that many other proteins relating to the ribosome structure and function were also detected in the TAP experiments, but they were not considered because they can be found in more than 3% of the successful protein purifications when this method is followed [34].

The experimental approach chosen to assess the role of this protein in ribosomal function also aimed to test whether a functional relationship with Sdo1p could be found, given the great structural similarity between these two proteins. The Yhr087wp protein shares with Sdo1p -and with other members of the family of proteins related to human SBDS- the FISH domain, characterized by a specific and highly-defined secondary structure: 2 β - 2 α - β - 2α. Prediction studies of the secondary and three-dimensional structure conducted by several authors [18,22] reveal that Yhr087wp is very similar to the Nt region of Sdo1p, where this domain is located. In evolutionary terms, Yhr087wp and Sdo1p have been proposed to have a relationship of a distant homology, and must result from a divergent evolution process [18]. The results presented in this work (Figure 8 and Table 3) indicate that, although Sdo1p is involved in translation and Yhr087wp could participate in this process, each one of these proteins would have its particular role in it, being that of Yhr087wp related with stress response. Actually Sdo1p is not regulated by adverse growth conditions [2].

Our studies done to determine the relationship of Yhr087wp with the ribosomal function provide some interesting results. On the one hand, the deletion mutant shows sensitivity to cycloheximide (and to hygromycin to a lesser extent), which also occurs in the mutants in proteins Scp160 and Tif4631p (Figure 7), both of which interact with Yhr087wp and have an unambiguous role in translation. On the other hand, the polysome profiles show that the *Δyhr087w* mutant is slightly more affected in translation activity than the wild type strain when high glucose osmotic stress is applied to exponentially growing cells. It is worth mentioning that our results indicate that the repression of translational activity described by Melamed et al. [37] under high salinity conditions also occurs with an osmotic stress due to 20% glucose, although during a shorter time.

The translation defect found in mutant *Δyhr087w* under 20% glucose does not result in a reduced translation of all the mRNAs and maybe for this reason the overall effect is low. Instead, some specific mRNAs involved in the stress response, such as those corresponding to *HSP104HSP78* and *GPD1*, are affected. It is noteworthy that the levels of translation of the *IPP1* or *PDA1* mRNAs (usually employed for normalization purposes in gene expression analyses under osmotic stress and growth in different carbon sources respectively [41,42]) are similar in both the wild type and mutant strains. On the other hand, our analyses also included *YGR052W* and *YJL107C*, two genes induced by osmotic stress [13-15]. In this case, and at the time considered, the P/FM ratio was higher in the mutant strain. The differences found with the other contemplated stress-related genes could be due to the function developed (which still remains unknown for *YGR052W* and *YJL107C*) or to the kinetic variations in the P/FM ratio during the translation recovery to the applied osmotic stress. It is important also to note that the cDNA levels corresponding to these genes are lower under the tested conditions than those found for the others considered.

The lower polysomal/free plus monosomal ratio found for the *HSP104* and *HSP78* mRNAs in the mutant strain, if compared with the wild type, is consistent with the lower levels encountered for these proteins ( Additional file 2: Figure S3) and with previous proteomic analyses [15]. Moreover, it can explain, together with the results found for *GPD1*, the growth defects displayed by that strain under high glucose stress [15]. The relationship found for YHR087wp between a role in translation recovery after osmotic stress treatment and loss of viability and growth reduction in its mutant strain has been recently observed for Cbc1p, which encodes the nuclear cap-binding protein [43]. Nevertheless, elucidating the particular role of each one of these proteins in translation recovery will require further analyses. The detection of the protein homologues of protein Yhr087wp in fungi (Table 4 and Additional file 2: Figure S4) suggests that this protein peforms a relevant role, particularly in stress response according to the data available from *Schizosaccharomyces pombe*[38]. Taking into account the function of Yhr087wp in the response to high sugar concentrations and the high mRNA levels found under these conditions we propose *HGI1* (High Glucose Induced 1) as a possible name to design the encoding gene in the future.

## Conclusions

Our results lead us to several conclusions: (1) Yhr087wp protein is distributed throughout the cell, independently of the growth conditions, and is partially found in the ribosomal fractions. (2) The expression of the coding gene is controlled by the transcription factors Msn2/4p, Sko1p and Hot1p in a way depending on the stress conditions considered, being particularly highly relevant the latter in the response to high glucose conditions. (3) Several experiments carried out in this work provide new insights about the involvement of this protein in the response to stress: it interacts with Cmk1/2p and Sti1p and displays a genetic relationship with the Bcy1p subunit of the PKA. (4) According to TAP experiments, it also interacts with several proteins participating in translation, including translation factors eIF4E and eIF4G. This result and the increased sensitivity of the deletion mutant strain to translation inhibitors points to a role of this protein in translation. (5) Under conditions of high glucose stress the translation recovery is quicker than when high salinity is applied. This recovery shows a certain delay in the *Δyhr087w* strain. (6) The ratio between the association to polysomal and free and monosomal fractions of mRNAs corresponding to several stress response genes ( *HSP104*, *HSP78* and *GPD1*) is reduced in the deletion mutant when compared with the wild type strain after the application of high sugar stress which indicates that the lower levels of some stress proteins in the mutant strain might be due to defects in their translation, and provides a possible explanation for the lower osmotic stress resistance described for this strain. The existence of homologues in other yeasts and fungi reinforces the relevance of this protein.

## Materials and methods

### Yeast strains, growth conditions and genetic methods

All the *S. cerevisiae* strains used for these experiments are shown in Additional file 1: Table S1. Growth of yeast strains without auxotrophies was carried out in YPD medium (1% (w/v) yeast extract, 2% (w/v) bactopeptone, 2% (w/v) glucose) at 30°C with orbital shaking (200 rpm). Strains with auxotrophies were grown in SC medium (0.17% (w/v) nitrogen base without amino acids and ammonium sulphate, 0.5% (w/v) (NH_4_)_2_SO_4_, 0.2% (w/v) *Drop out* mix without the selected amino acid or nucleobase, 2% (w/v) glucose). The solid medium also contained 2% (w/v) agar. In most experiments, liquid cultures were kept in the exponential growth phase for 16-24 h; then cells were treated under the required conditions; in the case of osmotic stress by high glucose concentration, cells were collected and transferred from YPD to the same medium containing 20% (w/v) glucose (YP20).

For the growth assays on resistance to osmotic stress or antibiotics, yeast cultures were diluted to the same OD_600_, and serial dilutions (1:10) were spotted onto YPD-derived plates, which were incubated at the desired temperatures. For the osmotic stress analyses plates contained 20%, 25% or 30% (w/v) glucose (YP20, YP25 and YP30) or 1 M KCl. YPD plates with cycloheximide (0.1 μg/mL) or hygromycin (10 or 20 μg/mL) were used for antibiotic resistance purposes. In some experiments, for a more accurate determination of the differences between strains, colony forming units were counted after application on the plates of the appropriate volumes and dilutions.

To construct *YHR087W* deletion strains, the wild-type coding sequence was replaced with the *URA3* gene through amplification of a disruption cassette from the YEp352 plasmid with oligonucleotides DELW-A and DELW-B ( Additional file 1: Table S2) and yeast transformation [44]. A similar procedure was used for *HOG1* gene deletion, with the utilization of the oligonucleotides DELHOG-A and DEL-HOG-B. The *YHR087W* gene was tagged with GFP in the FY86 strain using the Longtine method [45]; for this purpose, oligonucleotides YHR087W-F2 and YHR087W-R1 ( Additional file 1: Table S2) were used to amplify the transformation cassette from the pFA6a-GFP(S65T)-kanMX6 plasmid. To introduce the TAP tag into the same gene in strain FY86, a PCR amplification was carried out on the pBS2623 plasmid with oligonucleotides YHR087W-TAP L and YHR087W-TAP R. In the derived strain and in the corresponding wild type strain, genes *TIF32, CDC33, STI1* and *TIF4631* were independently tagged with HA. For this purpose oligonucleotides TIF32-F2, TIF32-R1, CDC33-F2, CDC33-R1, STI-F2, STI-R1 and TIF4631-F2 and TIF4631-R1 ( Additional file 1: Table S2) were used to amplify the transformation cassette from the pFA6a-3HA-HIS3MX6 plasmid [45]. Detection of Hsp104p in Western analyses was possible for the introduction of a HA tag by means of the primers HSP-F2 and HSP-R1. Selection of transformants by antibiotic resistance was achieved in YPD plates containing geneticin at a final concentration of 100 mg/L. Selection of transformants by auxotrophy was followed in SC medium plates without the corresponding amino acid or nucleobase.

### Gene expression analyses

For RNA isolation and quantification, a protocol explained elsewhere was followed [46]. cDNA was obtained as described previously [47]. The absence of DNA contamination in these preparations was assessed by analyzing the intron-containing gene *ACT1* by semi-quantitative RT-PCR using the oligonucleotides ACT-1 and ACT-2 shown in Additional file 1: Table S2 [17]. The *ACT1* gene was also used as a reference gene in some experiments given its constitutive expression under the conditions considered in this work [2,15]. cDNA preparations were used for the gene expression analysis by means of semi-quantitative RT-PCR or Real-Time RT-PCR as previously described [15].

Gene expression studies were carried out in some cases by Northern analyses, following the procedure described in Carrasco et al. [46]. Probes were obtained by PCR amplification of genomic DNA with the oligonucleotides described in Additional file 1 Table S2. In this case, normalization was carried out with gene *SCR1*, which is transcribed by the RNA polymerase III.

### Chromatin immunoprecipitation analyse

Cells from an exponential growth culture (OD_600_ 1) were incubated for 10 or 20 min (depending on the strain considered) under the stress condition (YP20 or 37°C). A control culture was kept for the same time in YPD. Cells were crosslinked with 1% (v/v) formaldehyde for 15 min at room temperature and were then incubated for 5 min with 125 mM glycine before being collected, washed with TBS buffer and frozen. Cells were resuspended in 300 μL of lysis buffer (50 mM HEPES-KOH pH 7.9, 40 mM NaCl, 1 mM EDTA, 1% (v/v) Triton X-100, 0,1% (w/v) sodium desoxicholate, 1 mM PMSF, 1 mM benzamidine and the *Complete Mini* protease inhibitor (Roche)) and 300 μL of glass beads were added. Cells were lysed for 30 min at 4°C in *Genie-2* (Scientific Industries). Chromatin was then fragmented by sonication in a *BioRuptor Diagenode* and the sample was centrifuged at 12000 rpm for 15 min.

To analyze polymerase binding to DNA, a cell extract was incubated with *Dynabeads Protein G* (Invitrogen) previously bound to an 8WG16 antibody (anti Rpb1, *Covance*) for 15 min at room temperature with orbital rotation. Beads were then washed three times with 200 μL de PBS 0.02% (v/v) Tween, and then once more with 100 μL of the same buffer. For the determination of Yhr087wp or Hot1p binding, a cell extract was incubated with *Dynabeads IgG Pan Mouse* (Invitrogen) with no antibody for 2 h at 4°C with orbital shaking. Then they were washed three times with 200 μL of PBS/BSA (5 mg/mL) and once more with 100 μL of the same buffer. Elution was carried out twice with 40 μL of 50 mM Tris–HCl pH 8.0, 10 mM EDTA, 1% (w/v) SDS, by heating at 65°C for 2 min at 600 rpm. In all cases, crosslinking was reverted by overnight incubation at 65°C with shaking. The eluted sample was digested for 90 min at 37°C with proteinase K 0.33 mg/mL and DNA was purified with the *High Pure PCR product purification kit* (Roche).

Co-immunoprecipitated DNA was analyzed in triplicate by Real Time RT-PCR in a *DNA Engine Peltier Termal Cycler* (Bio Rad) using the *Platinum SYBR Green qPCR SuperMix-UDG with ROX* (Invitrogen). Amplifications of two different regions of gene *HSP104* (5’ and 3’), and an intergenic region of chromosome V were carried out with combinations of oligonucleotides HSP104 3’ R/F, HSP104 5’ R/F, and Int A/B ( Additional file 1: Table S2). For the experiments of Hot1p binding, a region of the *YHR087W* gene promoter was amplified with the primers YHRPRO-2A and YHRPRO-2B. Data were processed with the ΔΔCT method [48] by comparing the results of the amplified from the immunoprecipitated sample (IP) with those of the whole cell extract (*Input*), and by using the intergenic region for normalization.

Semiquantitative PCR analyses were carried out as described by Alepuz et al. [33].

### Polysome analysis and ribosome preparation

Cultures from an overnight incubation in YPD up to an OD_600_ of 0.4 were transferred to YP20. The cells corresponding to 80 mL of the culture were collected before transfer (control condition) and at several times under stress and were incubated with 0.1 mg/ml of cycloheximide. Extracts were prepared according to the protocols described by Swaminathan et al. [49] and Garre et al. [43]. The volume corresponding to 10 units of A_260_ was applied to 10-50% sacarose gradients, containing KCl and MgCl_2_ concentrations of 140 mM and 5 mM, respectively, prepared with the *Density Gradient Fractionation System* (Teledyne ISCO). Samples were centrifuged at 35000 rpm for 2 h 40 min at 4°C, and were then scanned with the same system. Profiles were processed with the *Photoshop* software and the areas of the fractions were determined with *Image J* (NIH, *http://rsb.info.nih.gov/ij/)*. Gradient fractions were collected and processed for RNA isolation as described by Kuhn et al. [50], but including a precipitation with one volume of lithium chloride 5 M before the final precipitation with sodium acetate. The expression data for selected genes in the polysomal and free plus monosomal fractions were determined after normalization with the cDNA corresponding to *Bacillus subtilis LysA* mRNA transcribed *in vitro*: 96 ng of this mRNA were introduced in the ribosomal fractions used for RNA isolation. Finally, the expression data were relativized to the *ACT1* gene expression.

The ribosome fraction was obtained from yeast protein extracts by ultracentrifugación in a cushion of 25% glycerol in accordance with Meskauskas et al. [51]. For the electrophoretic analysis, the fraction corresponding to the whole cell extract was directly mixed with an equal volume of 2X SDS-PAGE solvent. Proteins of the non-ribosomal fraction were extracted by TCA precipitation and ribosomes were suspended in 50 mM Tris–HCl pH 7.5, 5 mM Mg(CH_3_COO)_2_, 50 mM NH_4_Cl, 0.1 mM PMSF, 0.1 mM DTT, 25% glycerol.

### Methods of protein manipulation and analysis

Protein extracts for routine analyses were prepared by resuspending cells in 200 μL of NaOH 0.1 M. Then they were kept at room temperature for 5 min and centrifuged for 1 min at 12000 rpm. The pellet was resuspended in 250 mM Tris–HCl pH 6.8, 140 mM SDS, 30 mM bromophenol blue, 27 μM glycerol, 0.1 mM DTT. After incubation at 95°C for 5 min, samples were centrifuged for 10 min at 3000 rpm and the supernatant was applied on the appropriate SDS-PAGE gel. TAP-tagged proteins were detected by Western analysis using the α-PAP antibody (Sigma) (dilution 1:2000 in PBS 0.1% Tween, 5% skim milk). For the HA-tagged proteins anti-HA 3 F10 was used (Roche) (dilution 1:10000 in PBS, 0.5% (v/v) BSA). α-tubulin antibody was obtained from GE Healthcare and was diluted 1:10000 in TBS 0.01% (v/v) Tween 20. The Rpl5 antibody (provided by Dr. J.L. Woolford) was used at the 1:5000 dilution in TBS, 0.01% Tween, 5% skimmed milk.

Fluorescent microscopy was performed in a *Zeiss Axioskop II* instrument using live cells grown in synthetic media with 2% or 20% glucose at up to an OD_600_ of 0.4. Nuclei were identified by a 30-min incubation at 30°C with Hoeschst reagent (*Invitrogen*) at a final concentration of 2 μg/mL. Images were obtained with a digital camera *SPOT* (Diagnostic Instruments Inc).

Tandem Affinity Purification (TAP) was carried out from the exponential cultures (6 L) of the strain containing the TAP-tagged version of Yhr087wp in YPD transferred for 90 min to YP20 (final OD_600_ of 2.0). Cells were collected, washed with water and frozen at -80°C. Afterwards they were disrupted with a *RETSCH MM301*, to finally obtain 30.42 g of lysed cells. Purification was carried out following the protocol described by Puig et al. [52], which involved two affinity chromatographies, one with IgG *Sepharose* and another with calmoduline. Finally, 6 fractions of 180 μL were obtained. The proteins associated with Yhr087wp were identified by mass spectrometry in the Proteomic Laboratory of the *Centro de Investigación Príncipe Felipe*. The information was analyzed by *Protein Pilot* ( *SwissProt*) and *MASCOT DAEMON* (NCBInr).

For the co-immunoprecipitation experiments, exponentially growing cells (OD_600_ of 1) were transferred for 90 min to YP20. The cells corresponding to 200 mL of these cultures were lysed in 150 μL of lysis buffer (50 mM Tris–HCl pH 8.0, 5 mM EDTA, 250 mM NaCl, 1 mM PMSF, 0.01% Triton X-100 and the *Complete* protease inhibitor mix (Roche)) in the presence of one volume of glass beads (425-600 μm in diameter). Samples were centrifuged at the maximun speed for 5 min. The supernatant obtained, which corresponds to the total protein extract, was then incubated with *Dynabeads IgG Pan Mouse* -prepared as described by the manufacturer ( *Invitrogen*)- for 2 h at 4°C in a rotary shaker to immunoprecipitate the Yhr087wp-TAP fusion protein. After collecting the non retained fraction, *Dynabeads* were washed 3 times with PBS buffer containing 5 mg/mL of BSA. Elution was carried out with 15 μL of 50 mM Tris–HCl pH 8.0, 10 mM EDTA, 1% SDS in a rotary shaker for 5-10 min at RT. The three fractions obtained were incubated at 95°C for 5 min in the presence of a protein solvent before being applied to a polyacrylamide gel at the appropriate concentration. Co-immunoprecipitated proteins were detected by Western with the anti-HA antibody 3 F10.

The BLAST tool of NCBI (http://blast.ncbi.nlm.nih.gov) was employed to search for homologous sequences. The sequences obtained were aligned by ClustalX 1.83 [53]. The alignment obtained was edited with Gblocks 0.91b [54,55].

## Competing interests

The authors declare that they have no competing interests.

## Authors’ contributions

MGA carried out most of the experiments and edited the manuscript. EJM performed experiments and edited the manuscript. MDO conceived the project, helped in several experiments, supervised experiments and wrote the manuscript. All authors read and approved the final version of the manuscript.

## Supplementary Material

Additional file 1 Yeast strains used in this work. Table S2: Oligonucleotides used in this work [7,56-60]. Click here for file

Additional file 2 Defects of the *YHR087W* deletion mutant under osmotic stress conditions. Figure S2: Relative binding of Yhr087wp to the 5’ and 3’ regions of the *HSP104* gene under conditions of osmotic heat shock stress determined by chromatin inmunoprecipitation experiments. Figure S3: Hsp104 protein levels in wild type and *Δyhr087w* strain. Figure S4: Sequence alignment between fungal protein homologues to *S. cerevisiae* Yhr087wp. Click here for file
