# Supplementary material for: The Saccharomyces cerevisiae Hot1p regulated gene YHR087W (HGI1) has a role in translation upon high glucose concentration stress
Source: BMC Mol Biol. 2012 Jun 21;13:19. doi: 10.1186/1471-2199-13-19 (PMC3441895; doi:10.1186/1471-2199-13-19)
Supplement: Additional file 1 — Yeast strains used in this work. Table S2: Oligonucleotides used in this work [7,56-60]. [file 1471-2199-13-19-S1.doc]

SUPPLEMENTARY TABLE 1. Yeast strains used in this work

|  | **Description** | **Origin** |
| --- | --- | --- |
| MCY1389 | *MATa*, *ura3-52, leu2::HIS3, SUC2* | F. Estruch (Estruch and Carlson, 1993) |
| BQS110 | MCY1389 *bcy1::LEU2* | F. Estruch (Estruch and Carlson, 1993) |
| FY86 | *MAT****a***, *ura3, his3, leu2* | Laboratory stock |
| BQS2071 | FY86 *YHR087W::GFP::kanMX4* | This work |
| TM141 | *MATa, his3Δ1, leu2Δ0, trp1Δ0, ura3Δ0* | F. Posas (Alepuz et al., 2003) |
| TM233 | TM141 *hog1Δ::TRP1* | “ |
| W303-1a | *MATa, ade2-1, trp1-1, can1-100, his3-11,15, leu2-3,112, ura3-1* | Thomas and Rothstein, 1989 |
| W303-1a *Δmsn2Δmsn4* | W303-1a *msn2Δ3::HIS3, msn4-1::TRP1* | F. Estruch (Estruch and Carlson, 1993) |
| W303-1a *Δhot1* | W303-1a *hot1:: kanMX4* | P. Alepuz (Alepuz et al., 2003) |
| BY4741 | *MATa, his3Δ1, leu2Δ0, met15Δ0, ura3Δ0* | Euroscarf |
| BY4742 | *MATα, his3Δ1, leu2Δ0, lys2Δ0, ura3Δ0* | Euroscarf |
| *Δyhr087w* | BY4742 *yhr087w::kanMX4* | “ |
| PAY575 | BY4741 *sko1::kanMX4* | “ |
| *Δsti1* | BY4741 *yor027w::kanMX4* | “ |
| *Δscp160* | BY4741 *yjl080c::kanMX4* | “ |
| *Δtif4631* | BY4741 *ygr162w::kanMX4* | “ |
| *YRP840* | *MATa leu2-3 112 his4-539 trp1 ura3-52 cup1::LEU2/PGK1pG/MFA2pG* | Hatfield et al., 1996 |
| *YRP1321 (cdc33^ts^)* | MAT**a** leu2 trp1 ura3 cdc33::LEU2 cup1::LEU2/PGK1pG/MFA2pG [cdc33-42/TRP1] | Schwartz and Parker, 1999 |
| *Δsti1 Δyhr087w* | *Δsti1* *yhr087w::URA3* | This work |
| *Δscp160 Δyhr087w* | *Δscp160* *yhr087w::URA3* | “ |
| *Δtif4631 Δyhr087w* | *Δtif4631* *yhr087w::URA3* | “ |
| *cdc33^ts^ Δyhr087w* | *cdc33ts yhr087w::URA3* | “ |
| *Δbcy1 Δyhr087w* | BQS110 *yhr087w::URA3* | “ |
| YHR087W-TAP | FY86 *YHR087W::TAP::kanMX4* | This work |
| FY86 STI1-HA | FY86 *STI1::HA::HIS6MX* | This work |
| YHR-TAP STI1-HA | YHR087W-TAP *STI1::HA::HIS6MX* | This work |
| FY86 TIF4631-HA | FY86 *TIF4631::HA::HIS6MX* | This work |
| YHR-TAP TIF4631-HA | YHR087W-TAP *TIF4631::HA::HIS6MX* | This work |
| FY86 TIF32-HA | FY86 *TIF32::HA::HIS6MX* | This work |
| YHR-TAP TIF32-HA | YHR087W-TAP *TIF32::HA::HIS6MX* | This work |
| FY86 CDC33-HA | FY86 *CDC33::HA::HIS6MX* | This work |
| YHR-TAP CDC33-HA | YHR087W-TAP *CDC33::HA::HIS6MX* | This work |
| HOT1-TAP | BY4741 *HOT1::TAP::HIS3* | Thermo |
| HOT1-TAP *Δhog1* | HOT1-TAP *hog1::URA3* | This work |
| SKO1-TAP | BY4741 *SKO1::TAP::HIS3* | Thermo |
| SKO1-TAP *Δhog1* | SKO1-TAP *hog1::URA3* | This work |
| Y16036 | BY4742 *cna1::kanMX4* | Euroscarf |
| *Δcna1Δyhr087w* | Y16036 *yhr087w::URA3* | This work |
| DAmP* TIF32 | BY4741 *tif32-kanR*-3’UTR | Thermo |
| DAmP TIF32 *Dyhr087W* | DAmP TIF32 *yhr087w::URA3* | This work |
| *tif32^ts^* | YKO^+^ derived *tif32::URA3* | Ben Aroya et al., 2008 |
| *tif32^ts^* | *tif32^ts^ yhr087w::kanMX4* | This work |

*Decreased abundance for mRNA perturbation. This strain belongs to a library created by Dr. Dr. Jonathan Weissman’s lab in which the 3’ untranslated region (UTR) of each essential gene present in the collection was disrupted with an antibiotic resistance cassette.

^+^Yeast Knock-Out collection (Yeast deletion project)

[Alepuz PM](http://www.ncbi.nlm.nih.gov/sites/entrez?Db=pubmed&Cmd=Search&Term="Alepuz PM"%5BAuthor%5D&itool=EntrezSystem2.PEntrez.Pubmed.Pubmed_ResultsPanel.Pubmed_DiscoveryPanel.Pubmed_RVAbstractPlus), [de Nadal](http://www.ncbi.nlm.nih.gov/sites/entrez?Db=pubmed&Cmd=Search&Term="de Nadal E"%5BAuthor%5D&itool=EntrezSystem2.PEntrez.Pubmed.Pubmed_ResultsPanel.Pubmed_DiscoveryPanel.Pubmed_RVAbstractPlus) E, [Zapater](http://www.ncbi.nlm.nih.gov/sites/entrez?Db=pubmed&Cmd=Search&Term="Zapater M"%5BAuthor%5D&itool=EntrezSystem2.PEntrez.Pubmed.Pubmed_ResultsPanel.Pubmed_DiscoveryPanel.Pubmed_RVAbstractPlus) M, [Ammerer](http://www.ncbi.nlm.nih.gov/sites/entrez?Db=pubmed&Cmd=Search&Term="Ammerer G"%5BAuthor%5D&itool=EntrezSystem2.PEntrez.Pubmed.Pubmed_ResultsPanel.Pubmed_DiscoveryPanel.Pubmed_RVAbstractPlus) G, [Posas](http://www.ncbi.nlm.nih.gov/sites/entrez?Db=pubmed&Cmd=Search&Term="Posas F"%5BAuthor%5D&itool=EntrezSystem2.PEntrez.Pubmed.Pubmed_ResultsPanel.Pubmed_DiscoveryPanel.Pubmed_RVAbstractPlus) F. 2003. Osmostress-induced transcription by Hot1 depends on a Hog1-mediated recruitment of the RNA Pol II. [EMBO](javascript:AL_get(this, 'jour', 'EMBO J.');) J. 22, 2433-42.

Ben-Aroya S, Coombes C, Kwok T, O'Donnell KA, Boeke JD, Hieter P. 2008. [Toward a comprehensive temperature-sensitive mutant repository of the essential genes of *Saccharomyces cerevisiae.*](http://www.ncbi.nlm.nih.gov/pubmed/18439903) Mol Cell. 30, 248-58.

Estruch F, Carlson M. 1993. Two homologous zinc finger genes identified by multicopy suppression in a SNF1 protein kinase mutant of *Saccharomyces cerevisiae*. [Mol. Cell. Biol](javascript:AL_get(this, 'jour', 'Mol Cell Biol.');). 13, 3872-3881.

Hatfield L, Beelman CA, Stevens A and Parker R (1996) Mutations in *trans*-acting factors affecting mRNA decapping in *Saccharomyces cerevisiae*. Mol Cell Biol, 16, 5830–5838.

Schwartz, D.C., Parker, R. 1999. Mutations in Translation Initiation Factors Lead to Increased Rates of Deadenylation and Decapping of mRNAs in *Saccharomyces cerevisiae*. Mol. Cell. Biol. 19, 5247-5256

Thomas BJ, Rothstein R. 1989. Elevated recombination rates in transcriptionally active DNA. Cell 56, 619-630.

SUPPLEMENTARY TABLE 2. Oligonucleotides used in this work

| Oligonucleotide | **Sequence (5’ to 3’)** | **Use** | |
| --- | --- | --- | --- |
| ADH1-A | AACGTTTCCGTTTCCGAAGC | | PCR of *ADH1* gene “ |
| ADH1-B | AAACCTCTGGCGAAGAAGTC | | “ |
| CLB2-UP | GGAAATAGCCGCCAAAAGAC | | PCR for Chromatin inmunoprecipitation experiments |
| CLB2-DOWN | CTGAAACTCTATGCCCATGC | | “ |
| CLN2-F | ATCTTTTTCGTATCCTCCGC | | PCR for Chromatin inmunoprecipitation experiments |
| CLN2-R | AAAGGGCCAACAGTTGTTTC | | “ |
| DELHOG-A | ACAAAGGGAAAACAGGGAAAACTACAACTATCGTATATAATAATTCGGTAATCTCCGAGC | | *HOG1* disruption |
| DELHOG-B | AAGAAGTAAGAATGAGTGGTTAGGGACATTAAAAAAACACGTCCCGGGTAATAACTGATA | | “ |
| HOG +200 | TGACGGTTCTTGGAGTCT | | Confirmation of *HOG1* disruption |
| HOG -200 | TGGCAGTGATGGACAGAT | | “ |
| URA3 UP | CTTAACTGTGCCCTCCAT | | Confirmation of *HOG1* disruption |
| DELW-A | GATAAAAATAAGAAAACCATCGCATACACAAAATAAAATCAAACATTCGGTAATCTCCGAGC | | *YHR087W* gene disruption with *URA3* |
| DELW-B | TATTTCAATGCAGGCTGTCCAGACAGAAGGAAAAGCTACCCCGGGTAATAACTGATA | | “ |
| DELYHR-F1 | GATAAAAATAAGAAAACCATCGCATACACAAAATAAAATCAACCGGATCCCCGGGTTAATTAA | | *YHR087W* gene disruption with *KanMX in tif32^ts^* |
| DELYHR-R1 | TATTTCAATGCAGGCTGTCCAGACAGAAGGAAAAGCTACGAATTCGAGCTCGTTTAAAC | | “ |
| YHR087W +200 | AATCATGTGTGACGAACG | | Confirmation of *YHR087W* gene disruption |
| YHR087W -200 | TGTCGAACTCTTCGAAGT | | Confirmation of *YHR087W* gene disruption |
| URA CHECK | GCGAAGAGCGACAAAGA | | ” |
| GPD1-1 | TTGAATGCTGGTAGAAAG | | PCR of *GPD1* gene |
| GPD1-2 | TGACCGAATCTGATGATC | | “ |
| HSP78-A | GTTGGGTGATGATGGTAAGA | | PCR of *HSP78* gene |
| HSP78-B | GCCAATCCTTCGCTTCATCA | | “ |
| HSP104-1 | GCGGTCTTACCGATACCTGG | | PCR of *HSP104* gene |
| HSP104-2 | GACTGAGCAGGCTCGTCAAGG | | “ |
| HSP104 3’ R | GATCCTTAGTGCCAGTTTGTTC | | Amplification of a 3’ region of *HSP104* gene |
| HSP104 3’ F | GAAATTGAAGAGAGATTCGAGC | | “ |
| HSP104 5’ R | AGATCATAGTCGTAACGGC | | Amplification of a 5’ region of *HSP104* gene |
| HSP104 5’ F | ATATGAACGACCAAACGC | | “ |
| HSP-F2 | ACGATAATGAGGACAGTATGGAAATTGATGATGACCTAGATCGGATCCCCGGGTTAATTAA | | HA-tagging of *HSP104* gene |
| HSP-R1 | AAACGTTAAAATGTGAGCTCTTTTGCTCGGGTGTCAAGTTCGAATTCGAGCTCGTTTAAAC | | “ |
| CHECK HSP 5’ | GAATGTCTGGAAGTTCTACC | | Confirmation of HA-tagging of *HSP104* |
| CHECK HSP 3’ | TTATCAACGCCATATGTCCC | | Confirmation of HA-tagging of *HSP104* |
| Int-A | GGCTGTCAGAATATGGGGCCGTAGTA | | Amplification of an intergenic region |
| Int-B | CACCCCGAAGCTGCTTTCACAATAC | | “ |
| IPP1-A | AAGAACAAGAAGTACGCTTTG | | PCR of *IPP1* gene |
| IPP1-B | TTGGAGCATCTGCCTTT | | “ |
| PDAQ-1 | CTCCTCCAGAAGCCAAAT | | PCR of *PDA1* gene |
| PDAQ-2 | CCTAGAGGCAAAACCTTG | | “ |
| STI-F2 | TTCAGACGTTGATCGCTGCTGGTATCATCCGGACTGGCCGCCGGATCCCCGGGTTAATTAA | | HA-tagging of *STI1*gene |
| STI-R1 | TGAGAAGATCATTATATGTACGTATGTATGAAAAAGCAGTAGAATTCGAGCTCGTTTAAAC | | “ |
| CHECK STI | GCTGCGATCATGCAAGATCCT | | Confirmation of HA-tagging of *STI1* gene |
| STI +100 | GATCCGGAGAACGACAGGAAA | | “ |
| TIF4631-F2 | ATATGTTCAGTGCATTAATGGGAGAAAGTGATGACGAAGAGCGGATCCCCGGGTTAATTAA | | HA-tagging of *TIF4631* gene |
| TIF4631-R1 | TTAGACTTTCTACCAACATCCTTGTATCCAAGTGACATTTTGAATTCGAGCTCGTTTAAAC | | “ |
| CHECK TIF4631 5’ | AAGAAATAGCCAAAGGGCTCC | | Confirmation of HA-tagging of *TIF4631* gene |
| CHECK TIF4631 3’ | GCACAGTAAGAGGATTACCCA | | “ |
| TIF32-F2 | CTGAAAAGTTGAGAGCCAAGAGATTGGCCAAGGGGGGCAGGCGGATCCCCGGGTTAATTAA | | HA-tagging of *TIF32* gene |
| TIF32-R1 | ACTGCCATCTTTACTGCTCTTCTGCTCCTTCCTTTGACATGGAATTCGAGCTCGTTTAAAC | | “ |
| CHECK TIF32 5’ | ATCGAACAGAGACTAGCC | | Confirmation of HA-tagging of *TIF32* gene |
| CHECK TIF32 3’ | AGTGCTCTTCCGCTAACG | | “ |
| CDC33-F2 | CCAGTGCCAATGGTAGACACCCTAACCATCAATCACCTTGCGGATCCCCGGGTTAATTAA | | HA-tagging of *CDC33* gene |
| CDC33-R1 | TGTTAACATGATGAGTTTATACGTGCATCCTAACTAGTAGAGAATTCGAGCTCGTTTAAAC | | “ |
| CHECK CDC33 5’ | TGCCTTATGGACTAAATC | | Confirmation of HA-tagging of *CDC33* gene |
| CHECK CDC33 3’ | GTCCTTTTAACGTCATCC | | “ |
| YGR052 R | TCTTGAATTAGGCAAGGTTTCTC | | PCR of *YGR052W* gene |
| YGR052 F | GTTTTGGGAACAGTGGTCTG | | “ |
| YHR087W-1 | GGTAAGCTATCTGAAGTTGTC | | PCR of *YHR087W* gene |
| YHR087W-2 | ACTTCTTCGATCTTCTTG | | “ |
| YHR087-F2 | TCAAAACCAAAGGGGGTAACGCCGGGAACCAAAGCCTACAATCGGATCCCCCGGGTTAATTAA | | GFP-tagging of *YHR087W* |
| YHR087-R1 | TTATGACAGGGCAGCACGCCAATTTTAACTTATTGCATATTTCAAGAATTTGCAGCTCGTTTAAAC | | “ |
| YHR check 5’ | TGAGTTCGGTAAGGGCAAGAA | | Confirmation of GFP-tagging of *YHR087W* |
| YHR check 3’ | TGTGTGACGAACGCGA | | “ |
| YHR087-TAP L | AGTCTCAAAACCAAAGGGGGTAACGCCGGAACCAAAGCCTACAATTCCATGGAAAAGAGAAG | | TAP-tagging of *YHR087W* |
| YHR087-TAP R | TGTTCTTTCTACTACTGTAAAAGAAGAATGCATGCGTTATGACAGGGCGACTCACTATAGGG | | “ |
| YHR CHECK 5’ | TGAGTTCGGTAAGGGCAAGAA | | Confirmation of TAP-tagging of *YHR087W* |
| OBS 292 | TTGTATTTTCAGGGTGAGCTC | | “ |
| YHRPRO-2A | GTCCCTTTCTGACAATAAGACC | | Chromatin inmunoprecipitation |
| YHRPRO-2B | TGTTTCTGGCGATCCCTTCG | | “ |
| YJL107C-1 | GAAACCTCCACATACGTACC | | PCR of *YJL107C* gene |
| YJL107C-2 | TGTTACAGCTTGTGAATCTG | | “ |
