# Supplementary material for: The Saccharomyces cerevisiae Hot1p regulated gene YHR087W (HGI1) has a role in translation upon high glucose concentration stress
Source: BMC Mol Biol. 2012 Jun 21;13:19. doi: 10.1186/1471-2199-13-19 (PMC3441895; doi:10.1186/1471-2199-13-19)
Supplement: Additional file 2 — Defects of the YHR087W deletion mutant under osmotic stress conditions. Figure S2: Relative binding of Yhr087wp to the 5’ and 3’ regions of the HSP104 gene under conditions of osmotic heat shock stress determined by chromatin inmunoprecipitation experiments. Figure S3: Hsp104 protein levels in wild type and Δyhr087w strain. Figure S4: Sequence alignment between fungal protein homologues to S. cerevisiae Yhr087wp. [file 1471-2199-13-19-S2.ppt]

## Slide 1
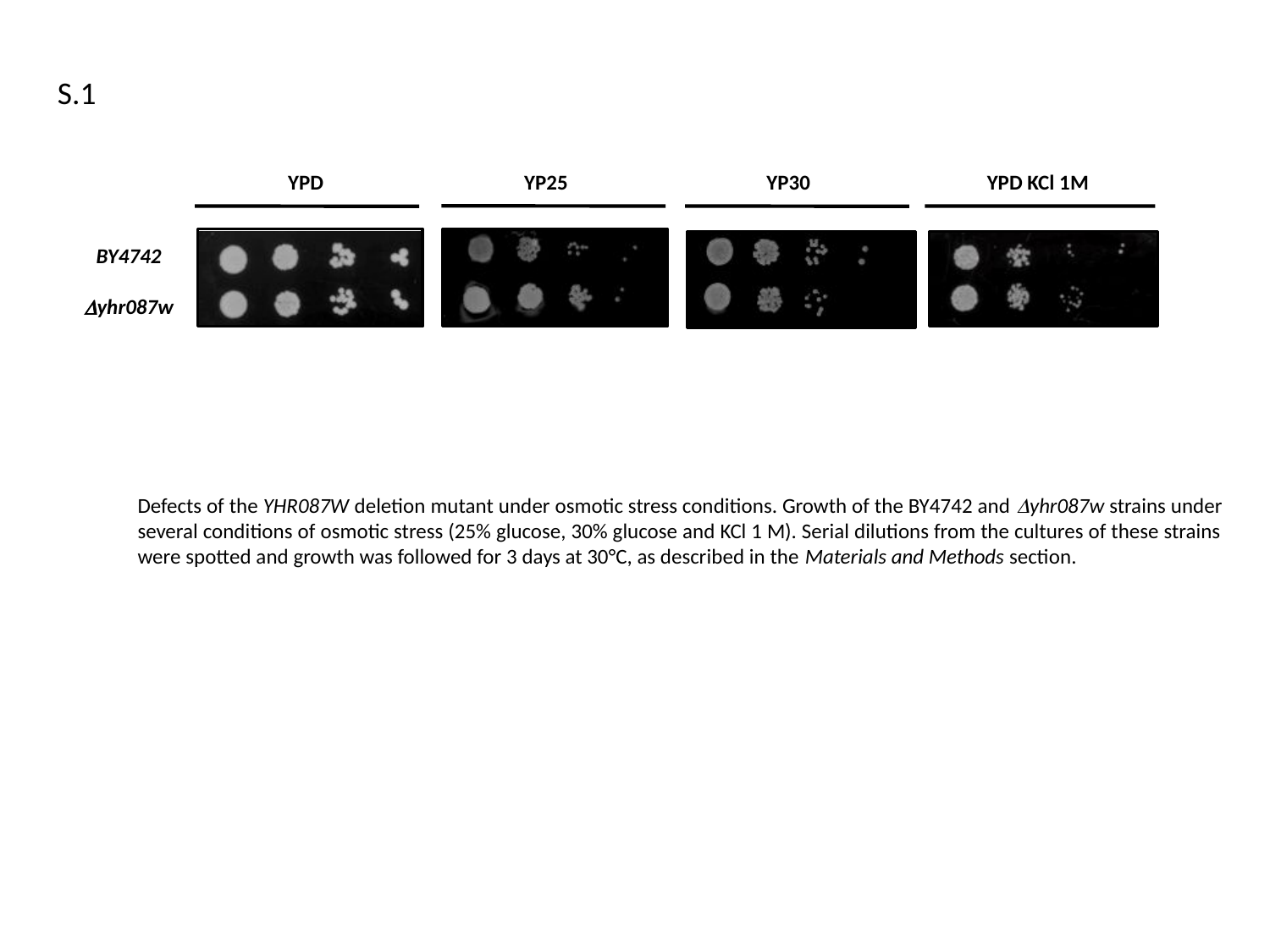

S.1
YPD
YP25
YP30
YPD KCl 1M
BY4742
Δyhr087w
Defects of the YHR087W deletion mutant under osmotic stress conditions. Growth of the BY4742 and Δyhr087w strains under several conditions of osmotic stress (25% glucose, 30% glucose and KCl 1 M). Serial dilutions from the cultures of these strains were spotted and growth was followed for 3 days at 30°C, as described in the Materials and Methods section.

## Slide 2
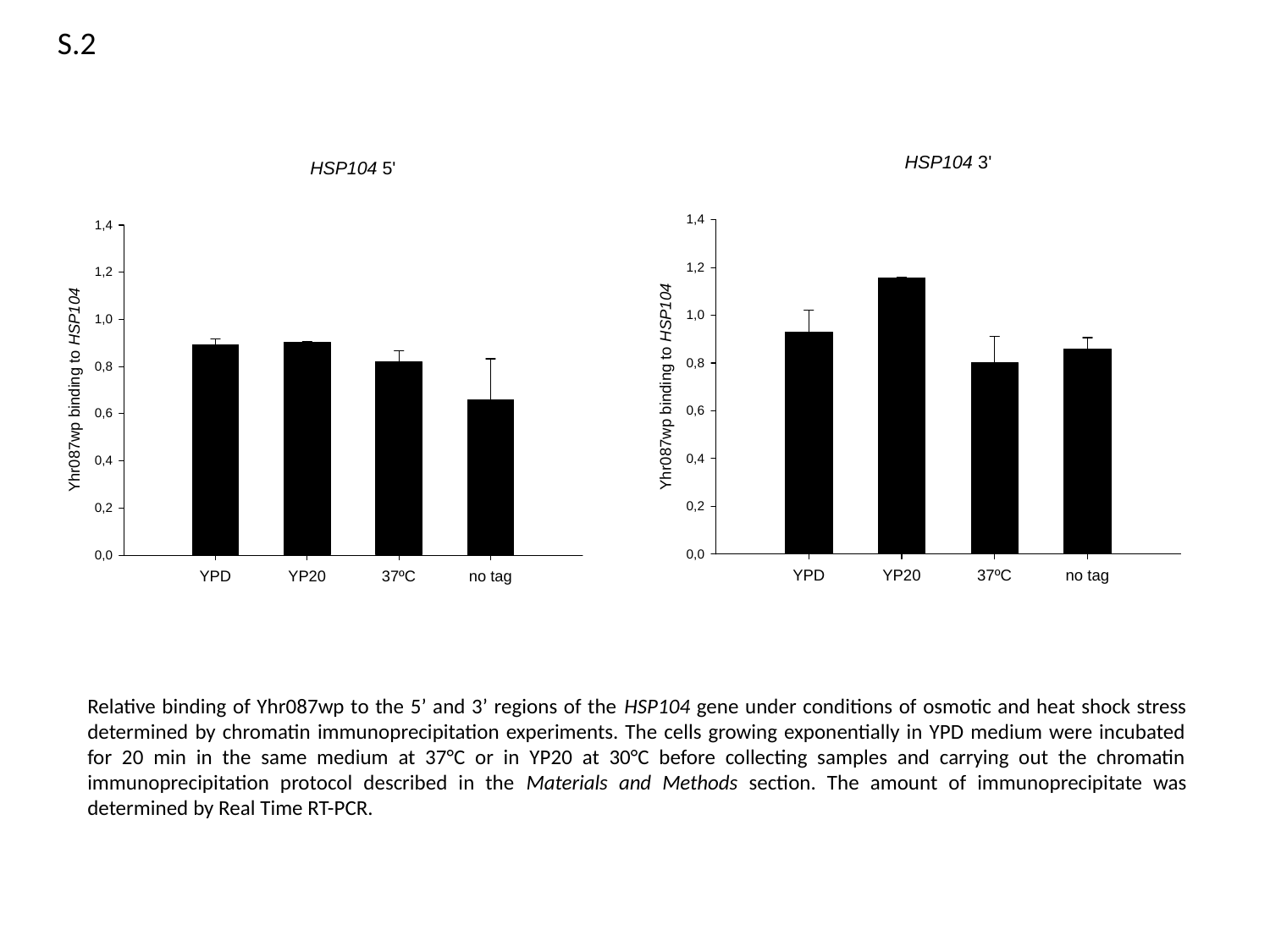

S.2
Relative binding of Yhr087wp to the 5’ and 3’ regions of the HSP104 gene under conditions of osmotic and heat shock stress determined by chromatin immunoprecipitation experiments. The cells growing exponentially in YPD medium were incubated for 20 min in the same medium at 37°C or in YP20 at 30°C before collecting samples and carrying out the chromatin immunoprecipitation protocol described in the Materials and Methods section. The amount of immunoprecipitate was determined by Real Time RT-PCR.

## Slide 3
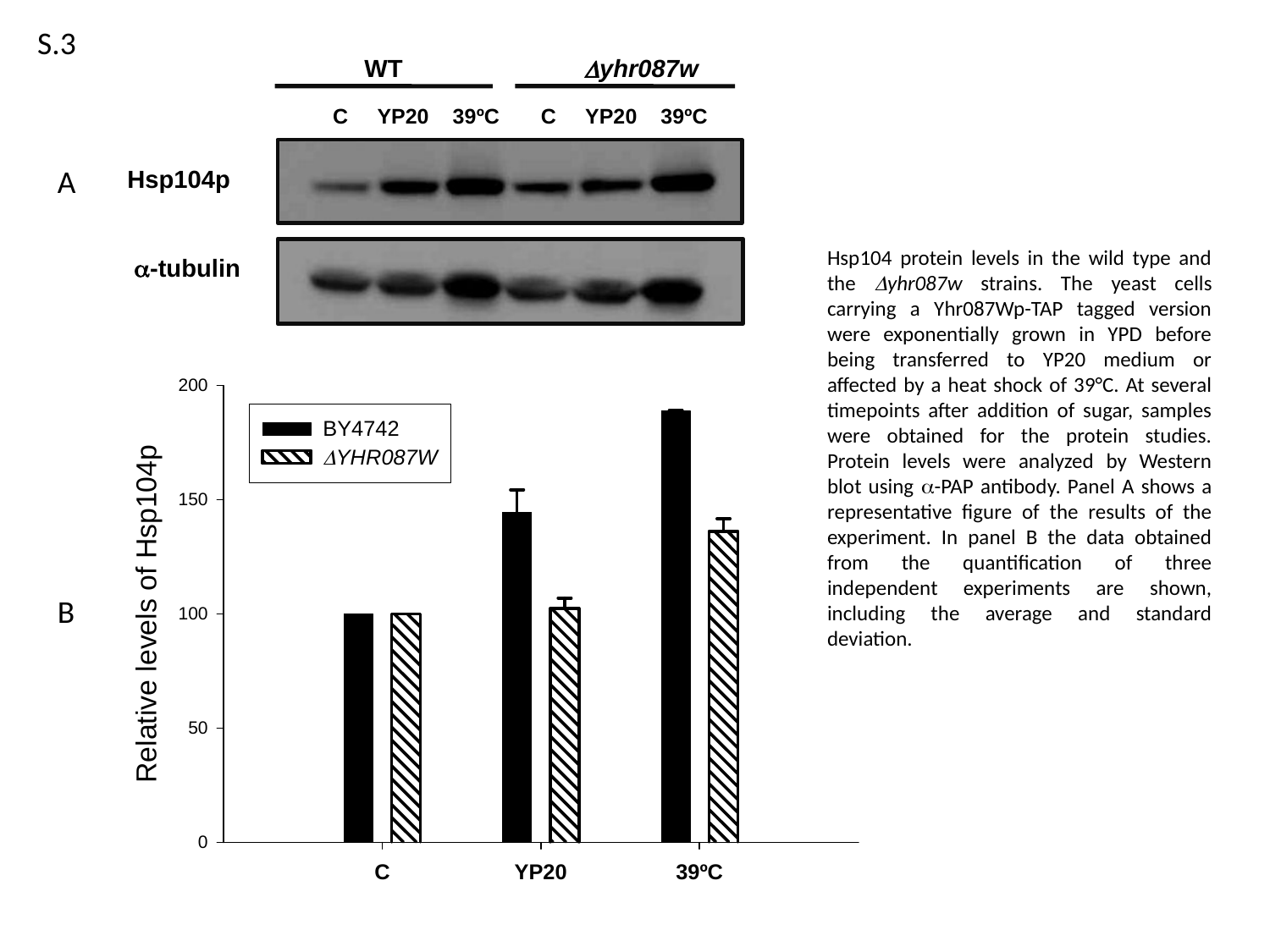

S.3
 	WT Δyhr087w
 C YP20 39ºC C YP20 39ºC
A
Hsp104p
 α-tubulin
Hsp104 protein levels in the wild type and the Δyhr087w strains. The yeast cells carrying a Yhr087Wp-TAP tagged version were exponentially grown in YPD before being transferred to YP20 medium or affected by a heat shock of 39°C. At several timepoints after addition of sugar, samples were obtained for the protein studies. Protein levels were analyzed by Western blot using α-PAP antibody. Panel A shows a representative figure of the results of the experiment. In panel B the data obtained from the quantification of three independent experiments are shown, including the average and standard deviation.
B

## Slide 4
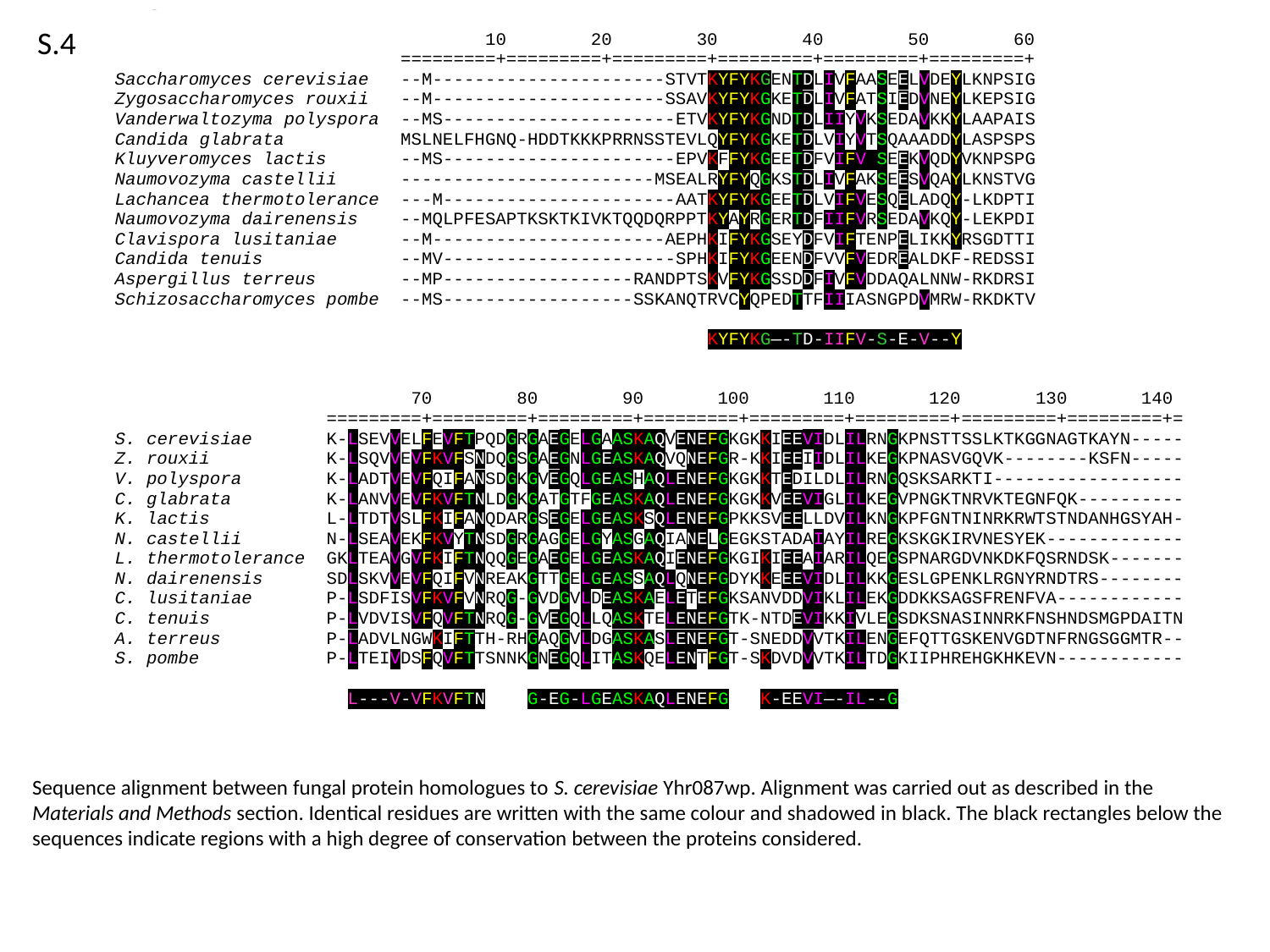

S.4
Sequence alignment between fungal protein homologues to S. cerevisiae Yhr087wp. Alignment was carried out as described in the Materials and Methods section. Identical residues are written with the same colour and shadowed in black. The black rectangles below the sequences indicate regions with a high degree of conservation between the proteins considered.
